# GoLoco/GPR motif-dependent regulation of Rap1GAP1 by Gα_o_ is disrupted by Gα_o_ encephalopathy variants

**DOI:** 10.1016/j.jbc.2025.110446

**Published:** 2025-07-02

**Authors:** Nathalie L. Momplaisir, Naincy R. Chandan, Beiyun Wang, Elaine Qu, Alan V. Smrcka

**Affiliations:** Department of Pharmacology, University of Michigan Medical School, Ann Arbor, Michigan, USA

**Keywords:** heterotrimeric G protein, alpha subunits, proximity labeling, protein-protein interactions, Rap1GAP, G protein-regulatory motif, BRET, GNAO1, encephalopathy, epilepsy, GoLoco motif

## Abstract

G protein-coupled receptors (GPCRs) that couple to Gα_i/o_ family members are major therapeutic targets. Among heterotrimeric G proteins, Gα_o_ is the most abundant Gα subunit in the brain, but the mechanistic pathways controlled by Gα_o_ have not been thoroughly established. Understanding Gα_o_-mediated signaling pathways is especially critical given recent reports of a neurodevelopmental disorder (*GNAO1* encephalopathy) associated with mutations in the Gα_o_-encoding gene. To address this gap, we sought to uncover novel Gα_o_ effectors using a proximity-based proteomics screen in differentiated PC12 cells. Our analysis revealed a diverse set of potential Gα_o_-GTP effector proteins, including a Rap1 GTPase-activating protein, Rap1GAP1. Regulation of Rap1GAP1 by G protein α subunits is controversial, with Rap1GAP1 reported to bind preferentially to Gα_o_-GDP *via* a GoLoco/G protein regulatory (GPR) motif. We establish that Gα_o_-GTP binds and regulates Rap1GAP1 activity and reveal a novel mechanism for Gα subunit recognition by Rap1GAP1 where the presence or absence of key contact residues in the GoLoco/GPR motif confer differential recognition of Gα_o_ guanine nucleotide binding status. We also show that pathologic *GNAO1* mutations disrupt this functional relationship by preventing the activated Gα subunit from attaining a conformation required for effector binding. These data resolve controversies in the literature regarding activation-dependent binding and regulation of Rap1GAP by Gα_o_ and help establish Rap1GAP1a as a bona fide G protein-regulated effector. Furthermore, our study finds that multiple mutants in Gα_o_ associated with *GNAO1* encephalopathy have defects in downstream effector interactions, which could underlie some of the manifestations of this disease.

G protein-coupled receptors (GPCRs) regulate a wide array of physiological processes through their ability to respond to diverse ligands, with significant impacts in both physiological and therapeutic contexts (1, 2). Upon activation, GPCRs relay extracellular signals by coupling to heterotrimeric G proteins, inducing structural rearrangements in the G protein that facilitate guanosine diphosphate (GDP)–guanosine triphosphate (GTP) exchange on the Gα subunit. This nucleotide exchange triggers the dissociation of the Gα subunit from its obligate Gβγ dimer, enabling both Gα and Gβγ to bind effector proteins and drive a plethora of cellular events (3, 4, 5, 6, 7).

The identity of the Gα subunit determines the classification of G proteins and specific mechanistic pathways mediated by GPCR activation. The Gα subunits are grouped into four major families: Gα_s_, Gα_i/o_, Gα_q/11_, and Gα_12/13_ (8, 9). The Gα_i/o_ family is the largest, comprised of the highly homologous Gα_i1_, Gα_i2_, Gα_i3_, Gα_oA_, Gα_oB_, Gα_t1_, Gα_t2_, Gα_gust_, and Gα_z_ (9, 10, 11). They are widely expressed across mammalian tissues, and GPCRs that couple to Gα_i/o_ subunits are among the most commonly targeted for therapeutic applications (2).

Gα_o_ accounts for approximately 1% of membrane protein in the central nervous system, making it the most abundant G protein in the brain (12, 13, 14, 15, 16). Recent studies have linked mutations in the gene that encodes for Gα_o_ (GNAO1) to early infantile epileptic encephalopathies/developmental epileptic encephalopathies, diseases whose mechanisms remain to be elucidated at the molecular level (17, 18, 19, 20, 21, 22). Gα_o_ couples to numerous neurotransmitter receptors, including muscarinic cholinergic receptors, GABAb receptors, α_2_-adrenergic receptors, and opioid receptors (23). Despite being discovered 4 decades ago and exhibiting prominent neuronal expression, the identity of effector proteins that couple directly to Gα_o_ remains unclear.

Most Gα_i/o_ family members suppress cyclic AMP (cAMP) production *via* direct adenylyl cyclase (AC) inhibition (3). However, Gα_o_ is unable to modulate AC enzymatic activity *in vitro* (24, 25). Nevertheless, activation of G_o_ by GPCRs inhibits cAMP in cell-based assays (22, 26), likely through a Gβγ-dependent mechanism (27). Early investigation of Gα_o_ function suggested regulation of calcium channels and potassium channels, but subsequent studies attributed these effects to the Gβγ subunit released from the G_o_ heterotrimer (28, 29, 30, 31, 32, 33). Identifying Gα_ο_ effectors has been challenging because tools that modulate Gα signaling also affect Gβγ, complicating the differentiation of subunit-specific signals.

Some proposed Gα_o_ effectors include the GTPase-activating protein for the Rap1 GTPase - Rap1GAP1, G protein-regulated inducer of neurite outgrowth (GPRIN), and Necdin (34, 35, 36, 37). These proposed effectors were identified using yeast two-hybrid systems. A more recent study used genomic and proteomic-based methods and identified Gα_o_-GTP as a Rab1 and Rab3 guanine dissociation inhibitor (GDI) displacer at the Golgi, regulating vesicular trafficking and neurite extension (38).

To elucidate the biological relevance of the Gα_o_ subunit and the mechanisms behind *GNAO1* encephalopathy, we utilized a recently developed unbiased proximity-based proteomic screening strategy previously used to identify novel interaction targets for Gα_i_ (39). This approach overcomes some of the limitations of conventional protein–protein interaction methods used to identify Gα effectors. Yeast two-hybrid assays lack appropriate cellular context, while affinity purification-mass spectrometry relies on detergent extractions that disrupt membrane-associated interactions (40). Thus, these methods fail to capture transient or compartmentalized effector interactions with Gα transducers. Consequently, multiple physiologically relevant Gα_o_ effectors likely remain unidentified.

Our proximity-dependent proteomic screen captured several previously reported Gα_o_ binding partners, including Gβγ and *GPRIN*. Rap1GAP1 (*RPGP1*) was the top Gα_o_-GTP enriched candidate. The identity of Rap1GAP as a Gα_o_ effector has been a subject of conflicting reports and, as a result, has led to controversy concerning its role as a Gαo-regulated effector. Initial studies reported that Rap1GAP preferentially interacts with Gα_o_-GDP to inhibit GAP activity (34). Follow-up studies found no binding of Gα_o_ to one splice variant of Rap1GAP1, Rap1GAP1a, but that an N-terminally extended splice variant, Rap1GAP1b, bound preferentially to Gα_o_-GDP, resulting in destabilization of Rap1GAP1b (41). Another laboratory found evidence for GTP-dependent interactions with Gα_i2_ and Gα_z_ but not Gα_o_ (42, 43, 44). Our identification of Rap1GAP1 as a top Gα_o_-GTP enriched candidate is inconsistent with Gα-GDP specific binding but is consistent with a recent report demonstrating Rap1GAP1a recruitment to the plasma membrane stimulated by Gi/o-coupled GPCRs (45). Here we show that Rap1GAP1a binds selectively to Gα_o_-GTP, while Rap1GAPb binds preferentially to Gα_o_-GDP. These differences are driven by variants of the N-terminal GoLoco/GPR (46, 47, 48) motif in Rap1GAP1 involving a novel mechanism for GTP-dependent G protein effector recognition *via* a truncated GoLoco/GPR motif. We also show that common *GNAO1* developmental epileptic encephalopathy variants cannot adopt the fully activated conformation required for Gα_o_-GTP binding to Rap1GAP1a.

## Results

### Proximity labeling by TurboID reveals multiple putative G**α**_o_ signaling interaction partners

To explore the Gα_o_ interactome, we leveraged a proximity-dependent approach to globally label endogenous potential interacting partners while retaining spatial compartmentalization and transient interactions networks (39, 49). We fused the promiscuous biotin ligase, TurboID, within the αb-αc loops of Gα_o_, a region known to tolerate internal tag insertions (50). We sought to detect both Gα_o_-GDP and Gα_o_-GTP-dependent interactions while minimizing confounding proximity-dependent bystander effects downstream of activated GPCR trafficking. For this purpose, we created a TurboID-Gα_o_^(Q205L)^ cDNA, where glutamine 205 was substituted with leucine to render Gα_o_ GTPase-deficient (51, 52) for comparison with wild-type Gα_o_ (TurboID-Gα_o_^(WT)^). To account for non-specific bystander enrichment of proteins by TurboID-Gα_o_ at the plasma membrane (PM), we used TurboID fused to the PM targeting sequence (CaaX) (TurboID-CaaX) derived from K-Ras as an additional control (Fig. lA).

Considering the prevalence of Gα_o_ in the central nervous system (15, 53), we set up the proximity labeling screen in neuronal-like differentiated PC12 cells to mimic the neuronal cellular environment. V5-tagged TurboID-Gα_o_^(WT)^, TurboID-Gα_o_^(Q205L)^, and TurboID-CaaX were introduced to differentiated PC12 cells using lentiviral transduction. After transduction, cells were labelled with biotin for 1 h, then lysed for streptavidin enrichment. Lysates were analyzed by SDS-PAGE and immunoblotting with a V5-antibody to test expression. Western blots show relatively equal expression of the TurboID-Gα_o_ and CaaX proteins (Fig. S1*A*). We then assessed the biotinylation across the transfection conditions for TurboID-Gα_o_ and TurboID-CaaX constructs *via* electrophoresis, followed by probing with streptavidin-IRdye800. In the absence of added biotin, minimal streptavidin labelling was observed, similar to cells transduced with YFP. In contrast, cells transduced with TurboID-fused constructs treated with exogenous biotin displayed enhanced streptavidin labelling (Fig. S1*B*). These results show that TurboID linked to Gα_o_ and CaaX effectively biotinylate proximal endogenous proteins.

**Figure 1:**
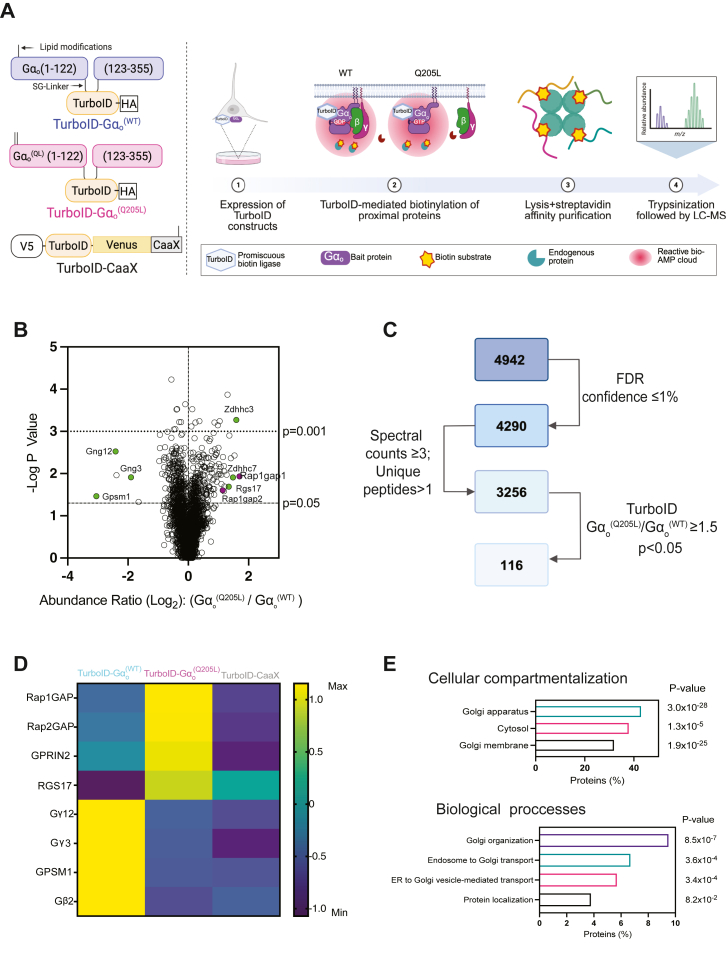
**Proximity labeling by TurboID reveals multiple putative Gα_o_ signaling interaction partners**. *A*, four days after differentiation with NGF, PC12 cells were transduced with lentiviral TurboID constructs fused to Gα_o_ and CaaX for 48 h. Biotin was then added to the cell media for 1 h followed by cell lysis. Biotin-labeled proteins were purified through streptavidin affinity capture and detected using tandem mass spectrometry. *B*, volcano plot depicting all identified proteins biotinylated by TurboID-Gα_o_^(WT)^ and TurboID Gα_o_^(Q205L)^. Dashed lines represent significant thresholds at *p*-values of 0.05 and 0.001. Previously reported interactors are labeled in green, while targets of interest are labeled in magenta. (N = 3). *C*, filtering strategy for proteins enriched by TurboID-Gα_o_^(Q205L)^. *D*, heat map depicting the relative abundance of reported Gα_o_ signaling interaction partners from the Turbo proximity labeling method. *E*, gene ontology analysis was carried out with DAVID Gene Ontology software to detect the prominent subcellular localization with associated biological processes of proteins that are preferentially biotinylated by TurboID-Gα_o_^(Q205L)^ [QL/WT ≥ 1.5; *p* < 0.05].

To capture relevant Gα_o_-signaling partners amongst the many proximally biotinylated proteins that were identified, we quantitatively compared differential enrichment of biotinylated proteins dependent on the nucleotide-bound state of the Gα subunit (Fig. 1*A*). Briefly, differentiated PC12 cells transduced with TurboID-CaaX, TurboID-Gα_o_^(WT)^, and TurboID-Gα_o_^(Q205L)^ were subjected to 1 h of biotinylation. Cells were lysed, and biotinylated candidates were captured on streptavidin magnetic beads. Biotinylated candidates across three biological replicates were quantified *via* liquid chromatography coupled to tandem mass spectrometry (LC-MS) (Fig. 1*A*). LC-MS analysis identified 4290 proteins with high confidence (false discovery rate (FDR) ≤ 1%) (Fig. 1, *B* and *C*). To identify potential active Gα_o_ interaction partners, the data were filtered to find proteins with a minimum peptide spectral match of 3, the number of unique peptides identified > 1, and a Gα_o_^(Q205L)^/Gα_o_^(WT)^ ratio ≥ 1.5 with a *p*-value < 0.05 (Fig. 1*C*). This analysis yielded 116 candidates enriched in Gα_o_^(Q205L)^ compared to Gα_o_^(WT)^ samples. Proteins showing Gα_o_-GDP enrichment were filtered by a Gα_o_^(WT)^/Gα_o_^(Q205L)^ ratio > 1.5 and a *p*-value < 0.05, resulting in 193 candidates (Fig. S2*A*).

The identities of many of the biotinylated proteins enriched in the TurboID-Gα_o_ samples align with previously reported Gα_o_ interactors (Fig. 1*D*). Multiple βγ isoorms and G protein signaling modulator 1 (GPSM1) (18, 54, 55) were among the top 10 TurboID-Gα_o_^(WT)^ enriched hits and are proteins known to interact preferentially with Gα_o_-GDP (Fig. 1*D*, Table 1). Gα_o_^(Q205L)^ enriched proteins included regulators of G protein signalling - RGS17 and RGS19, Rap1GAP, and Gα palmitoyl transferase enzymes - Zddhc3 and Zdhhc7 were within the top 10 ranked hits (34, 41, 56) (Fig. 1*D*, Table 2). It has been shown that these palmitoyl transferases isoforms are responsible for Gα_o_ palmitoylation in different compartments (57). RGS17 was previously identified to interact with Gα_o_ in a yeast two-hybrid screen (56).

**Table 1:** Top 10 TurboID-Gα_o_^(WT)^ enriched proteins in differentiated PC12 cells meeting the filtering criteria (WT/QL ≥ 1.5; *p* < 0.05; spectral counts ≥ 3; # unique peptides > 1)

Gene Symbol	Abundance ratio	*p*-value	Abundance ratio	
	QL/WT	QL/WT	QL/CaaX	CaaX/WT
*Gpsm1*	0.121	1.0 E-17	1.2	0.08
*Gng12*	0.188	1.0 E-17	1.5	0.12
*Gnao1*	0.192	1.0 E-17	0.93	0.21
*Gng3*	0.268	8.6 E-15	1.1	0.28
*Col1a1*	0.299	1.0 E-17	0.37	1.12
*Gpsm2*	0.309	1.8 E-15	0.95	0.27
*Ublcp1*	0.318	1.5 E-14	0.99	0.33
*Gnb2*	0.513	7.1 E-04	0.92	0.52
*Abcc1*	0.522	9.6 E-04	1.3	0.38
*Lamtor1*	0.523	1.0 E-03	0.21	2.47

**Table 2:** Top 10 TurboID-Gα_o_^(Q205L)^ enriched proteins in differentiated PC12 cells meeting the filtering criteria (QL/WT ≥ 1.5; *p* < 0.05; spectral counts ≥ 3; # unique peptides > 1)

Gene Symbol	Abundance ratio	*p*-value	Abundance ratio	
	QL/WT	QL/WT	QL/CaaX	CaaX/WT
*Rap1gap*	3.24	2.7 E-10	5.9	0.55
*Rgs19*	3.17	4.3 E-14	3.5	0.80
*Yipf3*	3.14	7.6 E-10	2.0	1.7
*Zdhhc3*	3.01	3.0 E-09	3.6	0.83
*NF1*	2.89	1.1 E-08	7.5	0.35
*Pdxdc1*	2.67	1.3 E-07	2.5	1.1
*Trip11*	2.57	3.6 E-07	1.5	1.7
*Rgs17*	2.53	5.4 E-07	1.4	1.9
*Slc30a6*	2.46	3.1 E-08	2.4	1.0
*Mon2*	2.41	2.0 E-06	2.5	1.1

Gene ontology (GO) analysis of Gα_o_^(Q205L)^ targets indicates strong enrichment for Golgi apparatus localization and vesicular transport (Fig. 1*E*). This is consistent with previous work showing a potential role of Gα_o_ in Golgi transport (38, 58). GO analysis of Gα_o_^(WT)^ targets revealed enrichment in plasma membrane and cytoplasmic localization in addition to the Golgi (Fig. S2, *B* and *C*).

### Rap1GAP1 shows preferential interaction with active G**α**_o_

**Figure 2:**
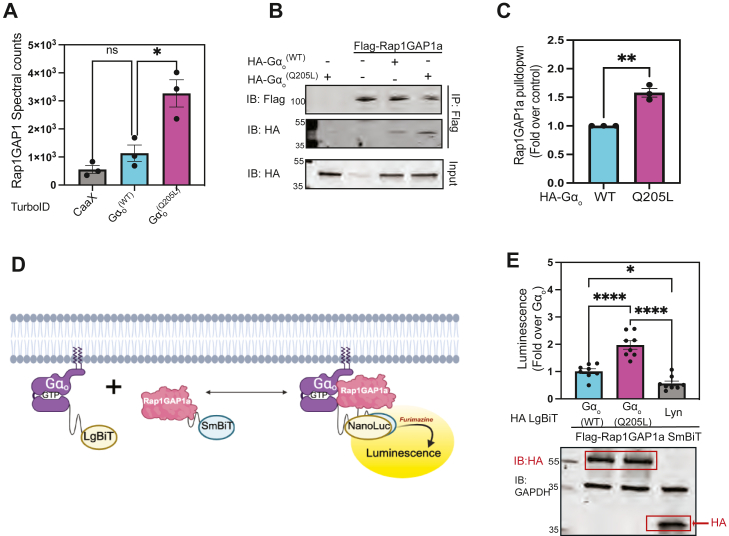
**RaP1GAP1a preferentially interacts with active Gα_o_**. *A*, normalized abundance (shown as mean ± SEM) of endogenous Rap1GAP1 detected by mass spectrometry in PC12 cells transduced with TurboID-Gα_o_^(WT)^, TurboID-Gα_o_^(Q205L)^, and TurboID-CaaX (N = 3) (∗*p* < 0.05, calculated using one-way ANOVA with Tukey's multiple comparisons test). *B*, cell lysates from HEK cells transfected with indicated constructs were subjected to immunoprecipitation with Flag antibody followed by gel electrophoresis and immunoblotting. Representative Western blot from 3 independent experiments shows Gα_o_ co-immunoprecipitated with Rap1GAP1a. *C*, quantification from three independent experiments (shown as mean ± SEM) of Gα_o_ pulled down with Rap1GAP1a (N = 3) (∗∗*p* < 0.01, calculated using an unpaired two-tailed *t* test). *D*, schematic of nanoluciferase-based complementation assay with C-terminally tagged Rap1GAP1a with SmBiT (Rap1GAP1a SmBiT) with Gα_o_ fused to LgBiT. *E*, HEK cells transfected with cDNAs encoding Rap1GAP1a SmBiT in conjunction with Gα_o_^(WT)^ LgBiT, Gα_o_^(Q205L)^ LgBiT, or Lyn LgBiT. 24 h after transfection, cells were transferred to a 96-well plate. 10 min after incubation with furimazine, luminescence generated from the complementation of the split-nanoluciferase pairs was measured. Data from eight independent experiments with a minimum of four technical replicates were plotted as mean ± SEM and analysed using one-way ANOVA with Tukey's multiple comparisons test (∗*p* < 0.05, ∗∗∗∗*p* < 0.0001). After the assay, cells were lysed and western blotted. A representative Western blot highlights the expression of all LgBiT-fused constructs.

The top Gα_o_^(Q205L)^ enriched candidate was Rap1GAP1 (Fig. 2*A*, Table 1). As discussed earlier, there is a lack of clarity as to whether Rap1GAP is a *bona fide* Gα_o_ effector, and the mechanisms of Gα_o_ regulation of Rap1GAP1 signaling are not well defined and controversial. Previous literature reported two major splice variants of Rap1GAP1 – Rap1GAP1a and Rap1GAP1b (47). They differ primarily in their N-terminus, a region that encompasses the GoLoco/GPR motif. Evidence from Uniprot (59) and GTEx (60) supports the existence of four major splice variants of Rap1GAP1 in the human genome. Sequence alignment of these variants is presented in Fig. S3. The first splice variant—Rap1GAP1a (P47736–1; ENST00000374765.9)—contains a truncated GoLoco/GPR motif. A second splice variant (P47736–2; ENST00000542643.6) also contains a truncated GoLoco/GPR motif but diverges in the C-terminal region. The third splice variant - Rap1GAP1b (P47736 3; ENST00000374761.6) contains a 33-amino acid extension at its N-terminus, resulting in a full consensus GoLoco/GPR motif. The last splice variant (P47736–4; ENST00000495204.5) contains a 64-amino acid extension at its amino terminus.

Our proteomic screen did not distinguish specific Rap1GAP1 variants enriched by Gα_o_-GTP. To clarify and understand Rap1GAP interactions with different nucleotide bound states of Gα_o_, we co-expressed full-length Flag-Rap1GAP1a with HA-epitope-tagged Gα_o_^(WT)^ and Gα_o_^(Q205L)^ in A293 cells. The Flag-M2 antibody was used to pull down Flag-Rap1GAP1a. The immunoprecipitated (IP) complex was resolved by SDS-PAGE followed by western Flag antibody to confirm successful pulldown of Flag-Rap1GAP1a, and with an HA antibody to detect co-precipitated Gα_o_. Rap1GAP1a interacted preferentially with Gα_o_^(Q205L)^ compared to Gα_o_^(WT)^ in this assay (Fig. 2, *B* and *C*). To further confirm this interaction, we used a nanoluciferase-based complementation assay (NanoBiT) (61, 62). Here, a small fragment of NanoLuc (SmBiT) was appended to the C-terminus of full-length Rap1GAP1a and the large subunit (LgBiT) was fused to the helical domain of Gα_o_ (61) (Fig. 2*D*). Co-expression of Gα_o_^(Q205L)^ LgBiT with Rap1GAP1a SmBiT yielded greater luminescence relative to Gα_o_^(WT)^ LgBiT, indicating a preferential interaction between Rap1GAPIa and Gα_o_-GTP (Fig. 2*E*). As a control for bystander interactions at the plasma membrane, Lyn-LgBiT transfected with Rap1GAP1a SmBiT resulted in significantly lower luminescence signal relative to both Gα_o_^(WT)^ and Gα_o_^(Q205L)^ LgBiT. Immunoblotting for HA-epitope tagged Gα_o_ and Lyn LgBiT revealed similar expression levels across all LgBiT-fused constructs (Fig. 2*E*).

### G**α**_o_ selectively recruits Rap1GAP1a to the plasma membrane

**Figure 3:**
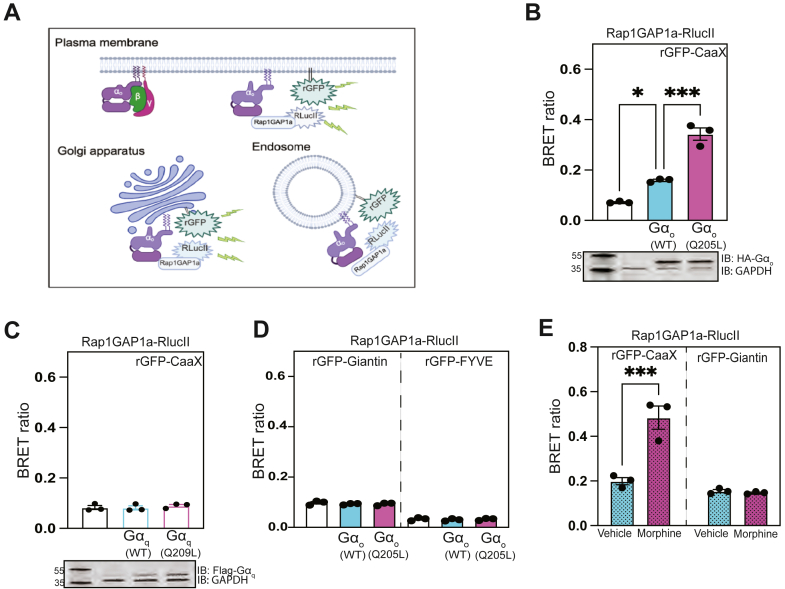
**Rap1GAP1a selectively interacts with Gα_o_ at the plasma membrane.***A*, schematic of enhanced bystander BRET approach to monitor interaction of Rap1GAP1a with Gα_o_ at distinct cellular compartments. *B*, the indicated plasmids were transfected in HEK cells. Twenty-four hours after transfection, cells were allowed to incubate in HBSS for 1 h. Following incubation, coelenterazine was added to each well. GFP2/RlucII ratios were calculated and plotted as means ± SEM for each condition (∗*p* < 0.05, ∗∗∗*p* < 0.001 calculated using one-way ANOVA with Tukey's multiple comparisons test). *C*, Rap1GAP1a PM translocation assayed in the presence of Gα_q_^(WT)^ and Gα_q_^(Q209L)^ using BRET (N = 3). *D*, Rap1GAP1a translocation assayed *via* BRET at the Golgi and endosome (N = 3). *E*, A293 cells were transfected with μ-opioid receptor, Gα_o_, Rap1GAP1a-RlucII with rGFP-CaaX or rGFP-Giantin. Forty-eight hours after transfection, cells were co-treated with vehicle or 100 nM morphine and coelenterazine400a. Data represent means ± SEM of three biological replicates performed in four technical replicates (∗∗∗*p* < 0.001, calculated using two-way ANOVA with Tukey's multiple comparisons test).

To delineate the spatial dynamics of Gα_o_-Rap1GAP interactions we adopted an enhanced bystander BRET (ebBRET) assay (45). In this assay, the BRET donor—a C-terminally truncated RAP1GAP1a construct (aa1-442) fused to *Renilla* luciferase (RlucII) was co-transfected with a BRET acceptor targeted to the plasma membrane—Renilla green fluorescent protein (rGFP) fused to CaaX (Fig. 3*A*). *Avet et al.* previously showed that dopamine D2R stimulation promoted ebBRET between Rap1GAP1a-RlucII and rGFP-CaaX in the presence of Gα_o_ (45). Similarly, we observed selectively enhanced recruitment of Rap1GAP1a to the plasma membrane with Gα_o_^(Q205L)^ relative to Gα_o_^(WT)^ (Fig. 3*B*). Rap1GAP1a-RlucII PM-translocation was not observed with Gα_q_-GDP or Gα_q_-GTP (Fig. 3*C*). At the receptor level, stimulation of the G_o_-coupled μ-opioid receptor (MOR) with DAMGO increased Rap1GAP1a-RlucII BRET at the PM; this was abolished by pertussis toxin (PTX) (Fig. S4*A*).

We also assessed recruitment of Rap1GAP1a to other membrane organelles. Transient co-expression of (1–442) Rap1GAP1a-RlucII with rGFP-Giantin (Golgi) or rGFP-FYVE (endosomes) and either Gα_o_^(Q205L)^ or Gα_o_^(WT)^ showed no Gα_o_-mediated recruitment of RapGAP1a to the Golgi apparatus, nor endosomes, respectively (Fig. 3*D*). Similar results were seen after stimulation of MOR with morphine (Fig. 3*E*). This was somewhat surprising given that Gα_o_ was expressed both at the PM and Golgi (Fig. S4*B*). In all, these findings provide evidence for the role of Gα_o_-GTP binding to Rap1GAP1a, facilitating Rap1GAP1a translocation primarily to the plasma membrane.

### The N-terminal region of Rap1GAP1 is a selectivity module for G**α**_o_

**Figure 4:**
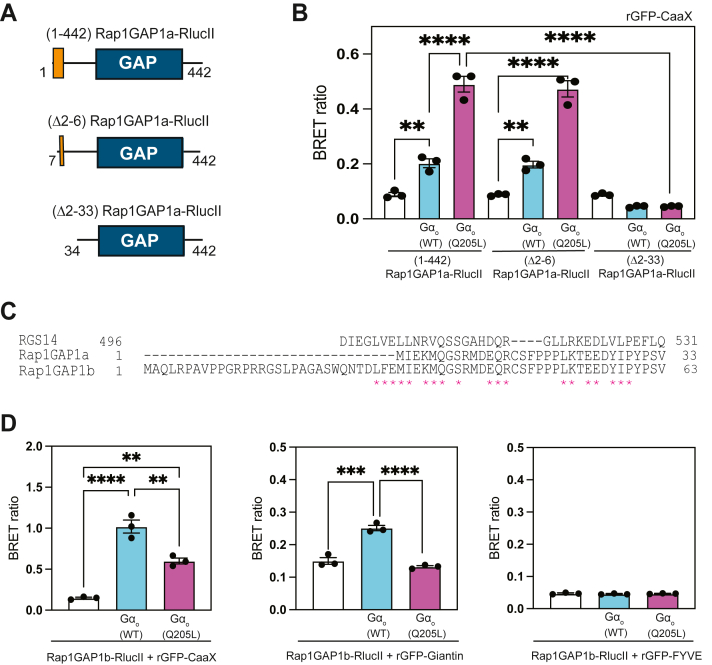
**The N terminus of Rap1GAP1 drives differential interaction with different nucleotide-bound states of Gα_o_**. *A*, schematic representation of Rap1GAP1a-RlucII constructs used in BRET assays, including N-terminal truncations. The GAP domain is highlighted in *dark blue* and the GoLoco motif in *orange*. *B*, A293 cells were co-transfected with Rap1GAP1-RlucII and N-terminal truncated constructs along with rGFP-CaaX and Gα_o_. BRET signal was measured 24 h after transfection following the addition of coelenterazine 400a. Data are shown as means ± SEM of three independent experiments (∗∗*p* < 0.01, ∗∗∗∗*p* < 0.0001, calculated using one-way ANOVA with Tukey's multiple comparisons test). *C*, sequence alignment of human Rap1GAP1a, Rap1GAP1b N-termini and the RGS14 GoLoco/GPR motif. *Stars* represent amino acids either identical or conserved relative to the RGS14 GoLoco/GPR motif. *D*, A293 cells were transfected with cDNAs encoding Rap1GAP1b-RlucII along with Gα_o_ and acceptors, rGFP-CaaX (*left panel*), rGFP-Giantin (*middle panel*), and rGFP-FYVE (*right panel*). ebBRET experiments were performed in three biological replicates, with each condition in four technical replicates. Data represent means ± SEM (∗∗*p* < 0.01, ∗∗∗*p* < 0.001, ∗∗∗∗*p* < 0.0001, calculated using one-way ANOVA with Tukey's multiple comparisons test).

To identify the molecular determinants that drive interactions of Rap1GAP1a with active Gα_o_, we started with truncations of the N-terminus of Rap1GAP1a-RlucII (1–442), which contains a partial GoLoco/GPR homology motif. We truncated the first 6 amino acids (Δ2-6 Rap1GAP1a), or the entire GoLoco/GPR motif (Δ2-33 Rap1GAP1a) (Fig. 4*A*). These constructs, fused to RlucII, were transfected into HEK cells with rGFP-CaaX and Gα_o_. Deletion of amino acids 2 to 6 of the GoLoco/GPR motif in Rap1GAP1a did not disrupt recruitment of Rap1GAP1a by Gα_o_-GTP to the PM (Fig. 4*B*). However, complete removal of the partial GoLoco/GPR motif in Rap1GAP1a abolished the ability of Rap1GAP1a to be recruited to the plasma membrane by Gα_o_^(Q205L)^ (Figs. 4*B*, S5*A*). These findings support the idea that the partial GoLoco/GPR motif in Rap1GAP1a is necessary for its engagement with active Gα_o_.

These results were surprising considering prior evidence of GoLoco/GPR motif-containing proteins in binding and stabilizing Gα_i/o_-GDP bound subunits (63, 64, 65, 66). This prompted us to assess the engagement of Rap1GAP1b with Gα_o,_ which harbors the full GoLoco/GPR motif (Fig. 4*C*). Co-transfection of Rap1GAP1b-RlucII with Gα_o_ shows preferential interaction with Gα_o_-GDP (Figs. 4*D*, S5*B*), consistent with previously reported findings (46, 47). Notably, selective recruitment of Rap1GAP1b by Gα_o_-GDP occurred both at the plasma membrane and the Golgi, in contrast to what was observed for Rap1GAP1a (Fig. 4*D*). These data show that differences in the N-terminal region of GoLoco/GPR motif in Rap1GAP1 dictate differential engagement with the nucleotide-dependent states of Gα_o_ at distinct cellular compartments.

### G**α**_o_ modulates the activity of Rap1GAPa at the plasma membrane

We then investigated whether Gα_o_ could modulate Rap1GAP1a activity. The standard approach for measuring Rap1 activation in cells uses cell extracts to affinity capture GTP-loaded Rap1 using beads pre-coupled with the Rap-binding domain of Ral guanine nucleotide dissociation stimulator (RalGDS) (67). However, this method disrupts the cellular context of the GTPase activation during cell lysis. A recent study circumvented this spatial limitation by using Förster Resonance Energy Transfer (FRET) to monitor RalGDS translocation to the plasma membrane in response to Rap1-GTP loading (68).

**Figure 5:**
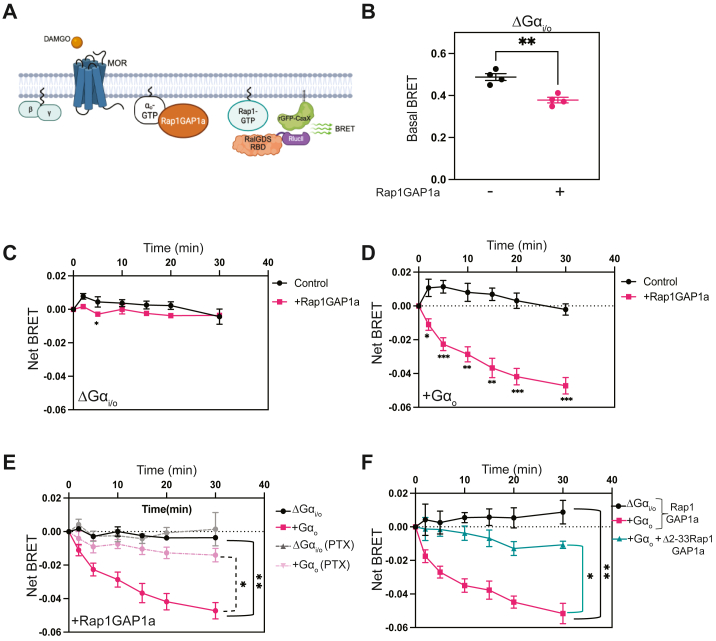
**Gα_o_ activates Rap1GAP1a activity**. *A*, diagrammatic representation of RalGDS translocation assay used to assess Rap1 activation at the plasma membrane. Cells were transfected with μ-opioid receptor, RalGDS-RlucII, rGFP CaaX, either with or without Rap1GAP1a. Upon Rap1 activation at the plasma membrane, RalGDS translocates to the plasma membrane. *B*, basal BRET signals were measured in Gα_i/o_ KO cells upon incubation with coelenterazine 400a. Data are represented as means ± SEM of 4 independent experiments, performed in four technical replicates (∗∗*p* < 0.01, calculated unpaired *t* test). *C*, assessment of RalGDS recruitment to the plasma membrane in Gα_i/o_ KO cells transfected with or without Rap1GAP1a stimulated with 10 μM DAMGO at time=0. Data are shown as means ± SEM of four independent experiments using two-way ANOVA with Sidak's multiple comparisons test, interaction of time X conditions (F6, 36), *p* = 0.2988. *D*, assessment of RalGDS PM recruitment in Gα_o_ transfected A293 cells. Forty-eight hours after transfection, cells were treated with 10 μM DAMGO at time=0 and BRET signal was measured. Data are shown as means ± SEM of four independent experiments using a two-way ANOVA with Tukey's multiple comparisons test, significant interaction of time X conditions (F6, 36), (*p* < 0.0001). *E*, A293 cells were co-transfected with indicated constructs. Twenty-four hours after transfection, cells were pre-treated with 100 ng/ml of PTX overnight. The next morning, BRET measurements were recorded. Data are represented as means ± SEM of four experiments using two-way ANOVA with Tukey's multiple comparisons test, significant interaction of time X conditions (F18,68), *p* < 0.0001). *F*, Rap1GAP1a GoLoco/GPR motif was validated as a determinant for regulation by Gα_o_. Briefly, RalGDS PM recruitment was assessed in DAMGO-stimulated Gα_i/o_ KO cells, Gα_o_-transfected A293 cells co-transfected with Rap1GAP1a and Δ2-33 Rap1GAPIa. Data are shown as means ± SEM of three experiments using two-way ANOVA with Tukey's multiple comparisons test, significant interaction of time X conditions (F12,36), *p* < 0.0001).

Building on this approach, we developed a RalGDS biosensor containing the RalGDS Rap binding domain, fused to *Renilla Luciferase* (RlucII) at the N-terminus (Fig. 5*A*). The N-terminus has been shown to tolerate GST fusion (69). First, we validated this biosensor in serum-starved A293 cells stimulated with epidermal growth factor (EGF). EGF promoted ebBRET between RalGDS-RlucII and rGFP-CaaX (Fig. S6*A*), consistent with previous reports showing RalGDS translocation to the PM following Rap1 activation (68, 70). RalGDS recruitment to the plasma membrane was abrogated by expression of Rap1GAP1a (Fig. S6*B*), confirming the specificity of this biosensor for Rap1-GTP.

To assess the role of Gα_o_ on Rap1GAP1a activity, we utilized Gα_i/o_ KO cells and transfected them with MOR, along with BRET pairs (RalGDS-RlucII and rGFP-CaaX) (Fig. 5*A*). Under basal conditions, membrane recruitment of RalGDS was suppressed in the presence of full-length Rap1GAP1a in Gα_i/o_ KO A293 cells (Fig. 5*B*). This indicates that these cells have a pre-existing pool of Rap1-GTP at the PM. Activation of MOR with DAMGO in these Gα_i/o_ KO cells did not alter RalGDS PM localization (Fig. 5*C*). Expression of Gα_o_ in these cells did not lead to significant MOR-dependent Rap1 inhibition, indicating that endogenous levels of Rap1GAP1a in A293 cells are not sufficient to support measurable Gα_o_ regulation of Rap1 activity. However, when Gα_o_ was cotransfected with Rap1GAP1a, DAMGO treatment resulted in a time dependent decrease in BRET, indicating inhibition of Rap1 activation at the plasma membrane through recruitment of Rap1GAP1a to the PM (Fig. 5*D*). This was blocked by PTX (Fig. 5*E*). Finally, we validated that MOR-stimulated Rap1 inhibition depended on Gα_o_ interaction with the partial GoLoco/GPR motif of Rap1GAP1a by expression of Rap1GAP1a missing the GoLoco/GPR motif (Δ2-33 Rap1GAP1a). Rap1 activity was only weakly inhibited upon stimulation with DAMGO in the Δ2-33 Rap1GAP1a-expressing cells (Fig. 5*F*). These data establish Gα_o_-GTP as an activator of Rap1GAP1a activity through its interaction with the GoLoco/GPR motif and recruitment to the PM.

### GNAO1 pathogenic mutants hinder Ga_o_-effector interaction

**Figure 6:**
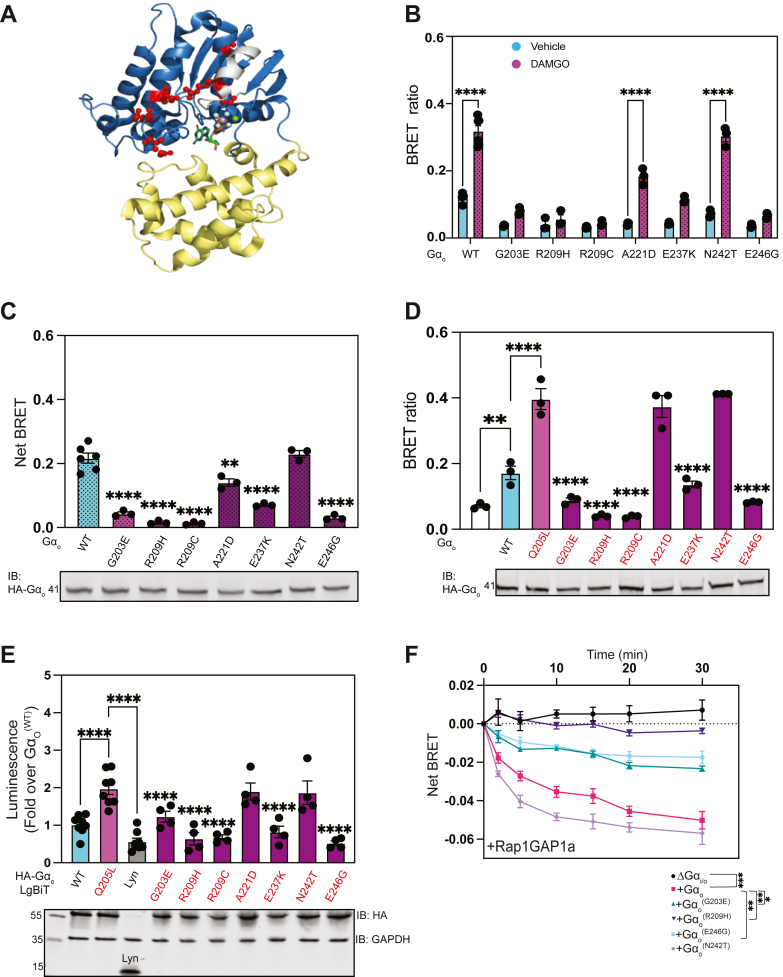
**GNAO1 encephalopathy causing mutants disrupt the functional interaction between Rap1GAP1a with Gα_o_**. *A*, diagram of a structure of Gα_o_ (PDB: 3C7K). The helical domain is highlighted in *yellow*, and the Ras-like GTPase domain is in *blue*, the switch II α2-helix highlighted in *gray*, AlF_4_^-^ is shown in *gray* and *blue spheres*, Mg^2+^ as a *greensph*, and *GNAO1*-causing encephalopathy mutants are indicated as *red spheres*. *B*, ebBRET ratio in A293 cells transfected with cDNAs encoding μ-opioid receptor, Rap1GAP1a-RlucII, rGFP-CaaX, Gα_o_ or Gα_o_ encephalopathy-causing mutants. Cells were treated with 10 μM DAMGO 48 h post-transfection to measure Rap1GAP1a recruitment. Data are shown as means ± SEM of 3 to 6 independent experiments, performed in four technical replicates (∗∗∗∗*p* < 0.0001, calculated using one-way ANOVA with Tukey's multiple comparisons test). *C*, agonist-induced net BRET for all mutants. Western blot depicts the expression of indicated Gα_o_ constructs. BRET measurements are obtained from a minimum of three independent experiments (∗∗*p* < 0.01, ∗∗∗∗*p* < 0.0001 calculated using one-way ANOVA with Tukey's multiple comparisons test). *D*, BRET ratio for A293 cells transfected with Gα_o_^(WT)^ and Gα_o_^(Q205L)^ or the indicated DEE Gα_o_^(Q205L)^ double mutants in red. BRET measurements are obtained from three independent experiments, performed in four technical replicates (∗∗*p* < 0.01, ∗∗∗∗*p* < 0.0001 calculated using one-way ANOVA with Tukey's multiple comparisons test). *E*, luminescence measurements from nanoluciferase complementation of A293 cells transfected with indicated constructs. Twenty-four hours after transfections, cells were replated in 96-wells and luminescence was measured following furimazine addition. All mutants labeled in *red* were introduced to the Gα_o_^(Q205L)^ construct. At least four independent experiments were performed. Data are represented as means ± SEM and all conditions were compared to Gα_o_^(Q205L)^ (∗∗∗∗*p* < 0.0001, calculated using one-way ANOVA with Dunnett's multiple comparisons test). *F*, recruitment of RalGDS to the plasma membrane assessed in HEK cells transfected with the indicated Gα_o_ constructs, BRET pairs, and the μ-opioid recep. Cells were stimulated with 10 μM DAMGO at time = 0. Data are shown as BRET ratios (∗*p* < 0.05, ∗∗*p* < 0.01, ∗∗∗*p* < 0.001, calculated using two-way ANOVA with Dunnett's multiple comparisons test, significant interaction of time X conditions (F30,102), *p* < 0.0001).

Characterization of Rap1GAP1a as an effector of activated Gα_o_ prompted us to explore the effects of *GNAO1* mutants on this interaction. We focused on a subset of *GNAO1* mutants located around switch II and III (Fig. 6*A*) that couple normally to receptors and exhibit minimal effects on Gβγ binding (17, 27). We reasoned that these mutants might be defective in downstream signaling due to impaired engagement between Gα_o_ and effectors.

To assess this, we monitored the effects of pathogenic *GNAO1* mutants on Rap1GAP1a PM recruitment in cells expressing MOR using ebBRET. We observed a drastic reduction in the ability of selected Gα_o_ mutants to recruit Rap1GAP1a-RLucII to the PM in response to DAMGO treatment, with the exception of Gα_o_^(A221D)^ and Gα_o_^(N242T)^ (Fig. 6, *B* and *C*). Interestingly, these defective mutants displayed lower basal BRET signal compared to Gα_o_^(WT)^ and had no basal constitutive activity. To differentiate these effects from defects in receptor coupling and Gβγ interactions, we examined this in the context of the Gα_o_^(Q205L)^ activating mutation. Rap1GAP1a PM recruitment in Gα_o_^(Q205L)^/Gα_o_^(Q205L/GNAO1 mutant)^ transfected cells mirrored those observed in DAMGO-stimulated cells. Gα_o_^(Q205L/A221D)^ and Gα_o_^(Q205L/N242T)^ retained the ability to recruit Rap1GAP1a to the PM, while other variants were defective (Fig. 6*D*). These results were corroborated by nanoBiT assays, confirming impaired binding interactions between Rap1GAP1a and these mutants (Fig. 6*E*).

We also tested the effects of these variants on Rap1GAP1a activity near the plasma membrane using the RalGDS translocation approach to measure Rap activity. In Gα_i/o_ KO cells expressing MOR, full-length Rap1GAPIa and Gα_o_^(N242T)^, RalGDS recruitment was suppressed after receptor activation, similar to Gα_o_^(WT)^ transfected cells (Fig. 6*F*). Interestingly, this mutant exhibited faster kinetics with significant differences observed around 10 min despite similar expression levels. Similar to our findings above (Fig. 6, *B*–*E*), Gα_o_ variants most frequently mutated (hotspot variants) in *GNAO1* encephalopathies (G203E, R209H, and E246 G) were ineffective at increasing Rap1GAP1a activity at the PM.

## Discussion

Downstream regulation of effector proteins through direct interaction with G proteins are major drivers that govern physiological processes downstream of GPCRs. In this study, we identified many known, and potential novel targets of Gα_o_ subunit transducers. Our objectives were to expand the current understanding of Gα_o_-mediated mechanistic pathways and understand how defects in Gα_o_-effector interactions may contribute to the mechanistic underpinnings of *GNAO1* epileptic encephalopathies (19).

### Identification of G**α**_o_ effectors

Gα_o_ is the most abundant G protein in the developing and adult brain, yet has a limited number of well-defined downstream molecular targets. Here, we used differentiated PC12 cells as a cell model for our search of Gα_o_ effectors. In the presence of nerve growth factor, PC12 cells adopt a neuronal-like phenotype and are an *in vitro* model for neuronal differentiation (71). Using a proximity biotinylation-based proteomic strategy, 4290 proteins were identified with high confidence, reflecting the broad scope of this approach and the requirement for quantitative filtering criteria to obtain meaningful protein-protein interaction data (72). Since G protein α subunits exist in two distinct nucleotide-bound conformations that have differential affinities for different binding partners, we reasoned that comparison of these states would enrich the data set for *bona fide* interaction partners. Thus, we compared the enrichment of each biotinylated target in TurboID-Gα_o_^(Q205L)^ to their corresponding TurboID-Gα_o_^(WT)^ abundance. This resulted in 193 Gα_o_^(WT)^-enriched candidates and 116 Gα_o_^(Q205L)^-enriched candidates for characterization. Among these filtered candidates were known interaction partners, including Gβ, Gγ, and activator of G protein signaling 3 (AGS3) in the inactive Gα_o_^(WT)^ samples (54, 55), and GPRIN2 and RGS17 in the Gα_o_^(Q205L)^ samples (73, 74). This reinforced the validity of our dataset and prompted our efforts to characterize potential novel Gα_o_ effectors identified in our screen.

Gene ontology analysis yielded a strong Golgi signature (*p*-value: 3.0 × 10^-28^) for Gα_o_^(Q205L)^-enriched candidates, compared to TurboID-Gα_o_^(WT)^ samples (*p*-value: 6.0 x 10^-4^), and is consistent with results from a previous screen for Gα_o_ binding partners (38). GO analysis ranked PM signaling as the most highly enriched signature for the TurboID-Gα_o_^(WT)^ samples, although some Golgi signature was observed. This QL-enrichment at the Golgi could be due to a higher affinity between Golgi-resident candidates with Gα_o_-GTP compared to Gα_o_-GDP or predominant localization of Gα_o_-GTP at the Golgi. While we did not assess Gα_o_ abundance at these compartments, our studies in PC12 and HEK cells confirmed its localization at both the plasma membrane (PM) and Golgi, consistent with previous reports in other cell types (18, 38, 57). A recent study investigated whether the PM pool of activated Gα_o_ is required for its activation at the Golgi (38). Although PM-endomembrane compartments of Gβγ shuttling has been reported (58, 75), activation of G_o_-coupled receptors does not induce Gα_o_ translocation from the PM to the Golgi (38). The Golgi-resident Gα_o_ subunit seems to be independently activated, and distinct functional properties have been associated with each compartmentalized Gα_o_ pool. The Gα_o_ pool at the Golgi has been proposed to cooperate with PM-localized Gα_o_ to regulate of neurite elongation and initiation, respectively (38). Our findings highlight a set of Golgi-localized Gα_o_ targets that have yet to be explored.

### Biochemical characterization of Rap1GAP isoforms with G**α**_o_

We focused on Rap1GAP1, a GTPase activating protein for Rap1 that regulates cell proliferation, cytoskeletal dynamics, and neuronal processes (41, 44, 70, 76, 77, 78, 79). At present, 12 transcripts for Rap1GAP1 with differential expression pattern in mammalian tissue have been reported in the Genotype-Tissue Expression (GTEx) portal (60). Upon searching the Uniprot database (59), we found 4 distinct translated variants of Rap1GAP1, adding on to the previous two variants that have been subject to investigation. Consistent with the literature, the major differences are observed in the N-terminal GoLoco/GPR motif region of Rap1GAP1, with some variants exhibiting divergence in their C-terminal region (Fig. S3). In our screen, the specific isoform enriched by TurboID- Gα_o_^(Q205L)^ could not be determined, since the identified biotinylated peptides were conserved among the variants. However, Rap1GAP1 transcripts with truncated GoLoco/GPR motifs (ENST00000374765.9; ENST00000374761.6) are highly expressed in the brain regions, including cerebellum, cortex, and nucleus accumbens. More specifically, the Rap1GAP1a encoding transcript (ENST00000374765.9) is the most enriched variant in adrenal gland—the tissue of origin for adrenal chromaffin cells, from which PC12 cells are derived. Given the source of PC12 cells and the abundance of Rap1GAP1a in both the adrenal gland and the brain, we reasoned the isoform enriched by TurboID-Gα_o_^(Q205L)^ to be Rap1GAP1a.

Using multiple approaches, we aimed to resolve discrepancies in the literature regarding Rap1GAP1 isoforms and their engagement with Gα_o_. One of the surprising findings of this study was our observation that differences in N-terminal GoLoco/GPR motifs of Rap1GAP1 underlie differential recognition by distinct nucleotide-dependent states of Gα_o_. Rap1GAP1 was first reported to bind Gα_o_-GDP (34); however, this interaction was not observed *in vitro* (41). Subsequent investigations revealed a frameshift mutation in the Rap1GAP1a construct, potentially contributing to the controversial data surrounding Rap1GAP1 and Gα_o_ (47). In our experiments, we found Rap1GAP1a to be the splice variant to selectively interact with Gα_o_-GTP. Rap1GAP1b, characterized by a full N-terminal GoLoco/GPR motif selectively interacts with Gα_o_-GDP.

**Figure 7:**
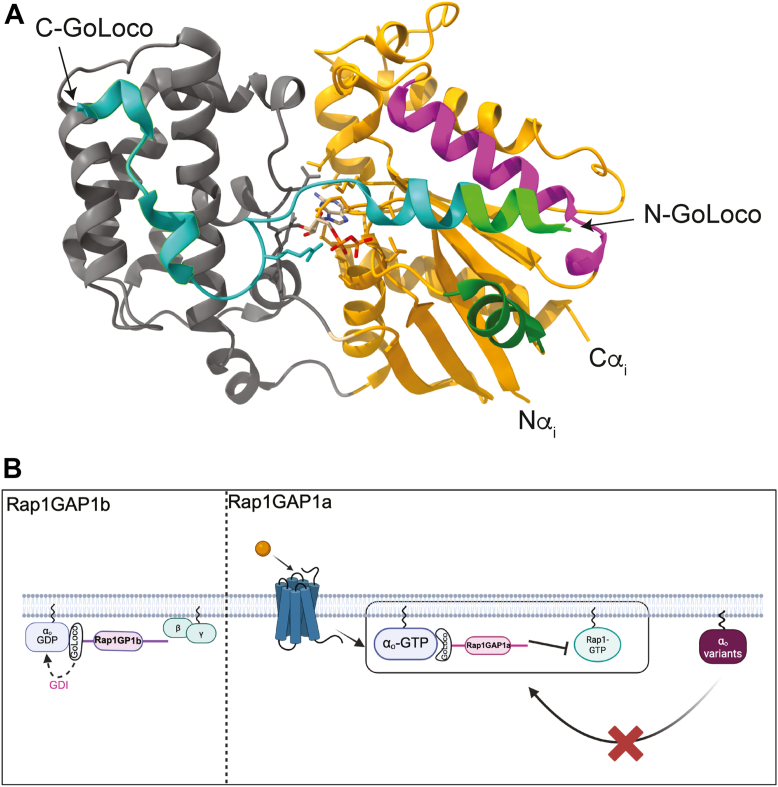
**Model of Rap1GAP1 interactions with, and regulation by Gα_o_ and Gα_o_-linked pathologic variants**. *A*, structural model of Gα_i1_ bound to the GoLoco/GPR motif from RGS14 (PDB: 3ONW). The RGS14 GoLoco motif is shown in *blue* and *light green*. The *light green* region represents amino acids analogous to the amino acids that are missing in the truncated Rap1GAP1a GoLoco motif. The α-helical domain from Gα_i1_ is in *gray* and the Ras-like domain is in *orange* with Switch II in *dark green* and the α-3 helix in *magenta*. *B*, Rap1GAP1 isoforms engage with Gα_o_ differentially. (*Left*) Rap1GAP1b preferentially engages with Gα_o_-GDP *via* a full GoLoco/GPR motif. (*Right*) Rap1GAP1a selectively engages with active Gα_o_ through its partial GoLoco/GPR motif. This results in reduced Rap1 activation at the plasma membrane. This functional interaction is impaired by pathogenic Gα_o_ variants.

GoLoco/GPR motifs canonically bind specifically to Gα_i/o_ subtypes and inhibit spontaneous GDP release and subsequent GTPγS association, categorizing them as G protein dissociation inhibitors (GDIs) (46, 63, 64, 65, 66). Although GDI activity was not assessed in this study, the absence of reported GDI activity by Rap1GAP1a on Gα_i/o_-GDP (47), along with our findings (Fig. 4), supports the idea that a full GoLoco/GPR motif is needed for Gα-GDP recognition. Crystallographic data with an RGS14 GoLoco peptide complexed with Gα_i1_-GDP show the N-terminal helix of RGS14 GoLoco engaged in a cleft between Switch II and the α3-helix in the GTPase domain in Gα_i1_-GDP (Fig. 7*A*). An arginine finger contacts the β phosphate of GDP to anchor it within the nucleotide-binding pocket. The C-terminus extends to contact the helical domain, effectively locking the Gα subunit in its closed GDP-bound state (46). When compared to Rap1GAP1b and RGS12/14, Rap1GAP1a lacks seven amino acids at the N-terminus of the GoLoco/GPR motif consensus and retains the putative arginine finger (47). This suggests the truncated GoLoco/GPR motif in Rap1GAP1a is missing residues essential for interaction with the Switch II-α3 cleft in the GTPase domain of Gα_o_-GDP, leading to loss of specific recognition of the GDP-bound conformation. How this loss enables the remainder of the motif to support specific recognition of Gα_o_-GTP by Rap1GAP1a is unclear. Further structural and mutagenic studies needed for an atomic-level explanation.

Our BRET assays also show distinct compartmentalization of these Rap1GAP1 isoforms. Selective Gα_o_-GDP-dependent recruitment of Rap1GAP1b was observed at both the plasma membrane and the Golgi. A prior study reported that Gα_o_ causes Rap1GAP1b to relocalize to a perinuclear region in Neuro2A cells (41). This perinuclear region might be correlated to the Golgi localization from our BRET assays. Rap1GAP1a, on the other hand, was restricted to the plasma membrane in the presence of Gα_o_-GTP, a pattern also observed with Gα_z_ (44). We did not examine other Gα_i_ family members in this study, but since it has been shown that the Rap1GAP1a-RlucII sensor detects GPCR activation of Gα_i1-3_ and Gα_z_ in ebBRET assays (45), it seems likely that other Gα_i_ family members differentially interact with Rap1GAP1 through mechanisms similar to those described here for Gα_o_.

We also observed robust activation of Rap1GAP1a GAP activity by Gα_o_-GTP at the plasma membrane that was dependent on the truncated GoLoco/GPR motif. This finding is particularly relevant considering previous studies demonstrating temporally controlled Rap1 signaling in ERK dynamics (68, 80, 81). Transient activation of ERK is linked to neuronal proliferation, whereas sustained ERK activation promotes neuronal differentiation (82). *Keyes et al.* have recently shown that Rap1GAP1a inhibits the sustained ERK pool at the PM, which in turn modulates protrusion dynamics in PC12 cells (68, 81). Since Rap1 signaling and Gα_o_ have shown to be implicated in neurite differentiation and axonal growth (44, 53, 83, 84), it is plausible that the localization of Gα_o_ at the cell periphery and its ability to regulate Rap1GAP1a at the PM contribute to ERK modulation at the PM. This, in turn, might likely influence actin-based processes critical for neuronal morphology and function.

### Influence of *GNAO1* associated mutations on G**α**_o_-Rap1GAP1a effector coupling

Building on these insights, we expanded our analysis to investigate the effects of *GNAO1* mutants on Rap1GAP1a-Gα_o_ interaction. To date, approximately 60 missense mutants in the *GNAO1* gene, encoding Gα_o_, have been reported in patients diagnosed with *GNAO1* encephalopathy. This class of neurodevelopmental disorders is accompanied by a range of motor disorders and/or epilepsy (85).

A recent report categorized these mutants by their ability to form the heterotrimeric G protein complex, to be activated by GPCRs, and to dissociate from Gβγ (17). We focused on a group of mutants, mostly located around Switch II-III regions, that exhibited seemingly normal association with the βγ subunit, with marginal effects on βγ dissociation. While defective interactions downstream the Gβγ subunit have been the main focus of previous *GNAO1* studies (17, 27), direct effector coupling downstream of the Gα_o_ subunit has yet to be investigated. Broadly speaking, hotspot mutations (G203E, R209H/C, and E246G) disrupt binding between Rap1GAP1a and Gα_o_. R209H exhibited near normal GPCR coupling and Gβγ dissociation; yet this mutant eliminates binding to Rap1GAP1a. This is independent of the localization of these mutants, as they exhibit both PM and Golgi localization in HEK cells (18). It has been suggested that these mutants are constitutively active based on BODIPY-GTPγS binding measurements. Our data indicate that except for Gα_o_^(N242T)^, these hotspot mutants fail to bind Rap1GAP1a or modulate Rap1GAP1a activity upon G_o_-coupled receptor activation indicating they are not constitutively activating for engagement of this effector system. Since these mutants dissociate from Gβγ upon receptor stimulation, they must be capable of binding GTP and undergoing changes at the Switch II-Gβγ interface associated with Gβγ dissociation. Thus, these mutants may be directly involved in contacts with effectors or result in failure to the switch regions on Gα to adopt the conformation required for effector engagement.

In conclusion, these findings expand the Gα_o_ interaction landscape and provide insights into Gα_o_-effector disruptions in *GNAO1* encephalopathy. We provide evidence that Rap1GAP is a true Gα_o_ effector, and that inconsistencies in prior studies can be explained based on the existence of multiple Rap1GAP isoforms have different preferences for the nucleotide bound state of the Gα subunit. Mechanistically, these preferences are driven by differences in the N-terminal GoLoco/GPR motif of Rap1GAP1 that determine recognition of Rap1GAP1 variants by either GDP or GTP bound Gα_o_ (Fig. 7*B*). Additionally, we demonstrate that common *GNAO1* encephalopathy-associated Gα_o_ variants cannot adopt the fully active conformation required for Gα_o-_GTP-dependent Rap1GAP1a engagement (Fig. 7*B*, right panel). Further characterization of the role of Rap1Gap1 and other novel Gα_o_-effectors in neurons is required to understand the physiological and pathophysiological roles of this and other Gα_o_-regulated effector systems.

## Experimental procedures

### Plasmid cDNA constructs

#### Proximity labeling

TurboID-CaaX: TurboID fused at N-terminus with a V5 epitope and to the C-terminus with mVenus, followed by the plasma membrane-targeting CaaX motif (KKKKKKSKTKCVIM, derived from the C terminus of K-Ras), was synthesized by GenScript. V5-TurboID was inserted into the helical region of Gα_o_, specifically between residues A^122^ and E^123^, and flanked on both sides by SGGGS linkers. The resulting V5-TurboID- Gα_o_ construct is Gα_o_ (1–122)- Linker-V5-TurboID-Linker- Gα_o_ (123–355).

#### Co-immunoprecipitation

2xFlag-Rap1GAP1a was obtained from Addgene (Plasmid #118324). Gα_o_ in pcDNA3.1+ was obtained from the cDNA resource center (cDNA.org). An HA epitope was inserted between A^122^ and E^123^ residues with SGGGS linkers flanking both regions using primers (Integrated DNA Technologies, IDT) and reagents and protocols in the Q5 site-directed mutagenesis kit (New England Biolabs, E0554S). The final construct is Gα_o_ (1–122)- Linker-HA-Linker-Gα_o_ (123–355). All missense mutations were generated using reagents and guidelines from the QuikChange II XL site-directed mutagenesis kit (Agilent,200,521).

#### Nanoluciferase complementation

2xFlag-Rap1GAP1a SmBiT was constructed as follows. The small subunit of the nanoluciferase SmBiT (VTGYRLFEEIL) was appendend to the C-terminus of Rap1GAP1a preceded by an SGGGGS linker using a Q5-site directed mutagenesis kit. The N-terminally targeted Lyn LgBiT (MGCIKSKRKDGRIPDI) in pTwist CMV BetaGlobin WRE backbone was synthesized by Twist Biosciences. Gα_o_ LgBiT was kindly provided by Julien Hanson's laboratory. An N-terminal HA tag was introduced similar to previous Gα LgBiT constructs (61) using the Q5-site-directed mutagenesis kit. Q205L mutation in the HA-Gα_o_ LgBIT construct, along with all other mutations in this construct were made with the QuikChange II XL-site-directed mutagenesis kit.

#### BRET biosensor constructs

The RlucII-tagged Rap1GAP1a construct (1–442 Rap1GAP1a-RlucII containing mutations to eliminate phosphorylation sites S437A, S439A and S441A), and all fluorescent acceptors, rGFP-CaaX (86), rGFP-Giantin, and rGFP-FYVE, were generously provided by the Bouvier laboratory from the Université de Montréal. Truncations within the GoLoco/GPR motif of Rap1GAP1a-RLucII (Δ2-6 Rap1GAP1a-RlucII and Δ2-33 Rap1GAP1a-RlucII) and extension of the N-terminus to create Rap1GAP1b-RLucII were performed using the Q5 Site-Directed Mutagenesis Kit. The N-terminally RlucII-fused RalGDS Rap-binding domain (RalGDS-RlucII) was synthesized by Twist Biosciences. The flag-tagged μ-opioid-receptor (MOR) clone in pcDNA3.1+ was generously gifted by the Puthenveedu laboratory from the University of Michigan. Gα_q,_ β_1_, and γ_2_ were all inserted in pcDNA3.1+ and obtained from the cDNA resource center. An internal flag tag between F^124^ and E^125^ was introduced to generate Flag-Gα_q_ WT. Q209 L mutation was introduced into Gα_q_-Flag-WT using a Quikchange mutagenesis kit.

### Cell culture

HEK cells (A293) were obtained from the American Type Culture Collection (ATCC). A293 cells were maintained in Dulbecco's modified Eagle medium (DMEM) (Corning, MT10013 CV) supplemented with 10% fetal bovine serum (FBS) (Gibco, 10,437,028) and 100 U penincilin/streptomycin (Gibco, 15,140,122) at 37 °C with 5% CO_2_. Cells were passaged using trypsin-EDTA (Gibco, 25,200,056). Transfections in HEK cells were conducted with lipofectamine 2000 (Invitrogen, 11,668,019) according to the manufacturer's protocol. HEK cells were discarded after passage 25.

Gα_i/o_ KO cells (87) were provided by the laboratory of Asuka Inoue from Tohoku University. These cells were cultured similarly to A293 cells, with the exception of using plates pre-coated with collagen IV (Sigma-Aldrich, C5533-5 MG).

### Reagents

The following primary antibodies were utilized: V5 (Cell Signaling Technologies, D3H8Q), rabbit anti-HA (Cell Signaling Technologies, C29F4), Flag (Invitrogen, PA1-984B and Cell Signaling, 14793S), mouse anti-flag (Sigma-Aldrich, F1804), Rap1GAP1 (Proteintech, 19174-I-AP), GAPDH (Invitrogen, MA5-15738), and mouse anti-GM-130 (BD Transduction Laboratories, 610,822). Streptavidin-IRDye800 was from LI-COR (925–32230). Antibodies were validated by comparing cells with and without transfection of the protein of interest and looking for immunoreactive bands at the correct molecular weight only present in the transfected cells. The following secondary antibodies were used: goat anti-rabbit DyeLight 800 (Invitrogen, SA535571), goat anti-rabbit IgG, 680 RD (LI-COR, 926–68071), goat anti-rabbit Alexa Fluor 568 (Thermo Fisher Scientific, A-11011), goat anti-mouse IRDye 800CW (LI-COR, 926–32210), goat anti-mouse IgG, 680 RD (LI-COR, 926–68070), and goat anti-mouse Alexa Fluor 488 (Thermo Fisher Scientific, A-11001). All secondary antibodies were diluted 10,000-fold and 1000-fold for immunoblotting and immunostaining purposes, according to the manufacturer's recommendations.

### Proximity labeling using TurboID coupled to mass spectrometry in PC12 cells

#### Small-scale total protein biotinylation and western detection

PC12 cells were seeded in Collagen IV-coated 6-well plates at a density of 0.5 × 10^6^ cells per well on Day 0. After 24 h, cells were treated with 100 ng/ml NGF to induce neurite outgrowth (Day 1) (Gibco, 13,257,019). Fresh media containing 100 ng/ml NGF was replenished 48 h later (Day 3). The following day (Day 4), cells were transduced with lentiviral supernatants carrying TurboID-Gα_o_^(WT)^, TurboID-Gα_o_^(Q205L)^, or TurboID-CaaX. After 24 h, fresh media supplemented with 100 ng/ml NGF was added to the cells (Day 5). On Day 6, cells were incubated with 500 μM biotin (Sigma-Aldrich) for 1 h, washed with ice-cold PBS, and lysed with 300 μl of 1X Laemmli buffer per well. The lysates were collected and heated at 95 °C for 10 min, and 40 μl of each sample was resolved on a 4 to 20% Mini-protean TGXTM Gel (Bio-Rad, 4,561,094).

#### Protein biotinylation, pull-down, and western detection

Low-passage PC12 cells were utilized for proximity labelling experiments and all experiments were performed in triplicate transfections performed on separate days (biological replicates). Cells were plated into Collagen IV-coated 175 cm^2^ flasks at a density of 5.5 × 10^6^ cells per flask. Differentiation and expression of TurboID fused proteins were performed following the protocol described above (Days 1–5). On Day 6, the media was replaced with 35 ml of DMEM containing 500 μM biotin and 10% FBS for 1 h. Immediately after incubation, the labelling medium was decanted, and the cells were washed twice with 1 × PBS, scraped from the flasks, followed by centrifugation at 4000*g* for 10 min. Cell pellets were washed twice with 1X PBS. The supernatant was aspirated, and the cell pellets were snap-frozen and stored at −80 °C until further use.

For streptavidin pull-down experiments, all stock solutions were freshly prepared except the lysis buffer. Low-protein binding tubes (Eppendorf, 022,431,081) were used for sample preparation. Frozen cell pellets were lysed in 1 ml of ice-cold lysis solution (modRIPA buffer: 50 mM Tris, 150 mM NaCl, 0.1% SDS, 0.5% Sodium deoxycholate, 1% Triton X-100, final pH 7.5) supplemented with 1X protease inhibitor (PI) cocktail (P8849, Sigma) 1 mM phenylmethylsulfonyl fluoride (PMSF) (G-Biosciences, 786–055) for 10 min on ice. The lysates were further incubated with 125 units of Benzonase (Sigma, E1014–25KU) in an end-over-end rotator at 4 °C for 20 min. Subsequently, 0.3% SDS was added to the lysates, which were incubated for an additional 10 min at 4 °C. The lysates were then centrifuged at 15,000*g* for 15 min, and the supernatant was transferred to fresh tubes. Protein concentrations were equalized using the Pierce 660-nm protein assay reagent (Thermo Fisher Scientific, 22,660).

A 5% aliquot of the equalized lysates was reserved for Western blot analysis to confirm biotinylation of input samples. The remaining lysates were incubated with 500 μl of Pierce streptavidin magnetic bead slurry (Thermo Fisher Scientific, 88,817) per sample under end-over-end rotation at 4 °C for 18 h. The beads were washed sequentially: twice with modRIPA buffer, and once with each of the following solutions - 1 M KCl, 0.1 M Na_2_CO_3_, 2% SDS (prepared in 50 mM Tris, pH 7.5), and 2 M Urea (prepared in 10 mM Tris, pH 8.0). Finally, the beads were washed twice with 1X PBS, snap-frozen, and stored at −80 °C until further processing for mass spectrometry.

### Protein digestion and TMT labeling

On-bead digestion followed by LC-MS/MS analysis was conducted at the mass spectrometry-based Proteomics Resource Facility of the Department of Pathology at the University of Michigan, as previously described (88). Samples were first reduced (10 mM DTT in 0.1 M TEAB at 45 °C for 30 min), then alkylated (55 mM two-chloroacetamide at room temperature (RT) for 30 min) in the dark. Following alkylation, proteins were digested with trypsin (Promega, V5113) at a 1:25 enzyme-to-protein ratio at 37 °C with constant mixing on a thermomixer. Proteolysis was stopped by the addition of 0.2% TFA, followed by desalting using a Sep-Pak C18 cartridge (Waters Corp, WAT036945). Desalted peptides were dried using a Vacufuge and reconstituted in 100 μl of 0.1 M TEAB. Each sample was labelled for 1 h using the TMT10plex isobaric labeling kit (Thermo Fisher Scientific, 0,090,110) according to the manufacturer's protocol.

The labeling reaction was quenched by adding 8 μl of 5% hydroxylamine and incubating for 15 min. The samples were then combined and dried. Offline fractionation of the combined sample into 8 fractions was carried out using a high pH reversed-phase peptide fractionation kit, according to manufacturer's protocol (Pierce, 84,868). Fractions were dried and reconstituted in 12 μl of 0.1% formic acid/2% acetonitrile for LC-MS/MS analysis. Sample-to-TMT channel information is provided in Table 3.

**Table 3:** TMT channel Assignment for TurboID samples in LC-MS/MS analysis

Biological replicate	Sample	TMT channel
1	Gα_o_-TurboID-WT	126
1	Gα_o_-TurboID-Q205L	127N
1	TurboID-CaaX	128N
2	Gα_o_-TurboID-WT	129N
2	Gα_o_-TurboID-Q205L	130N
2	TurboID-CaaX	131
3	Gα_o_-TurboID-WT	127C
3	Gα_o_-TurboID-Q205L	128C
3	TurboID-CaaX	129C

### LC-MS analysis

Liquid chromatography-tandem MS (LC-MS) with tandem mass tag (TMT) labeling was conducted by the Proteomics Resource Facility at the University of Michigan, as previously described (88). MS data were acquired using an Orbitrap Fusion (Thermo Fisher Scientific) coupled with an RSLC Ultimate 3000 nano-UPLC (Dionex). Briefly, 2 μl of each fraction was resolved on a nano-capillary reverse phase column (PepMap RSLC C18 column, 75 μm i.d. × 50 cm; Thermo Scientific) at a flowrate of 300 nl/min using 0.1% formic acid/acetonitrile gradient system (2–22% acetonitrile in 110 min; 22–40% acetonitrile in 25 min; 6 min wash at 90% followed by 25 min re-equilibration). Samples were directly sprayed onto the Orbitrap Fusion using EasySpray source (Thermo Fisher Scientific). MS1 scans were acquired by the mass spectrometer (Orbitrap; 120K resolution; AGC target 2 × 10^5^; max IT 50 ms). This was followed by data-dependent, “Top Speed” (3 s) MS2 scans (collision-induced dissociation; ion trap; NCD 35; AGC 5 × 10^3^; max IT 100 ms). To enhance quantitation accuracy, the top 10 precursors from each MS2 were fragmented by HCD for multinotch-MS3, followed by Orbitrap analysis (NCE 55; 60K resolution; AGC 5 × 10^4^; max IT 120 ms, 100–500 m/z scan range).

To analyse TurboID-MS data, proteome Discoverer (v2.4; Thermo Fisher) was used by searching the SwissProt rat database. The analysis parameters included MS1 tolerance of 10 ppm and MS2 tolerance of 0.6 Da. Static modifications were set for carbamidomethylation of cysteines (57.02146 Da) and TMT labeling of lysine and N-termini of peptides (229.16293 Da). Variable modifications included oxidation of methionine (15.9949 Da) and deamidation of asparagine and glutamine (0.98401 Da). Proteins and peptides with a false discovery rate threshold ≤1% were selected for further analysis. TMT ion reporter quantitation was based on MS3 spectra with an average signal-to-noise ratio of 10 and < 50% isolation interference.

### TurboID-MS normalization and sorting criteria

Because TurboID relies on proximity, we anticipated that many proteins identified in the screen would be labelled due to proximity to TurboID-fused constructs rather than direct interaction. As such, most proteins were expected to display similar levels of biotinylation across all TurboID Gα_o_^(WT)^ and TurboID Gα_o_^(Q205L)^. To address this, first we filtered the data by selecting proteins with high confidence, displaying a false discovery rate (FDR ≤ 1%). Next, we normalized protein abundances to account for variation across biological replicates in all groups (TurboID-CaaX, TurboID-Gα_o_^(WT)^, and TurboID-Gα_o_^(Q205L)^). This was achieved by summing the raw total abundance for each sample and normalizing them to the sample with the highest TMT signal, which in this case was TurboID-Gα_o_^(WT)^. Following this, a subsequent set of filters were applied to identify the relevant Gα_o_-interactome: Peptide Spectral Matches (PSM) ≥ 3, number of unique peptides identified > 1, abundance ratio TurboID-Gα_o_^(Q205L)^/TurboID-Gα_o_^(WT)^ ≥ 1.5, and abundance ratio *p*-value < 0.05.

### Gene ontology analysis

Gene ontology (GO) analysis was performed using the DAVID Bioinformatics resource at https://david.ncifcrf.gov. The criteria outlined in Figures 1*C* and S2*A* were used to establish a reference list of all proteins with a false discovery rate (FDR) ≤ 1% from our TurboID-MS screen, enabling the identification of biological processes enriched in TurboID-Gα_o_^(Q205L)^ and TurboID-Gα_o_^(WT)^ proteins relative to this reference list. The filtered candidates were analyzed by functional annotation clustering.

### Co-immunoprecipitation

A total of 1.5 × 10^6^ of HEK - A293 cells was seeded into poly-D-lysine (Sigma, P6407-5 MG) 10 cm-dishes (Fisherbrand, FB012924) and incubated overnight at 37 °C with 5% CO_2_. The next day, plasmids encoding 1 μg Flag-Rap1GAP1a, 2 μg HA-Gα_o_^(WT)^, and 2 μg HA-Gα_o_^(Q205L)^ were transfected into these cells using Lipofectamine 2000 at a 1:3 DNA to lipofectamine ratio. After 24 h, cells were washed once with cold 1X Phosphate Buffer Saline (PBS) (Fisher, BP3994) and replaced with 500 μl 1X modRIPA lysis buffer (50 mM Tris-HCl pH 7.5, 150 mM NaCl, 0.5 mM EDTA, 1 mM EGTA, 1 mM MgCl2, 1% Nonidet-P40 (NP40), 0.1% sodium dodecyl sulfate, 0.4% sodium deoxycholate, protease inhibitor cocktail (Sigma-Aldrich, P8340)), diluted 100-fold and phenylmethylsulfonylfluoride (PMSF) (Sigma, 7626-5G) diluted-1000-fold. Cells were incubated with modRIPA on ice with mild agitation. Cells were then scraped, and lysates were transferred to a centrifuge tube and incubated on ice for 30 min with occasional vortexing. Lysates were then pelleted by centrifugation at 13,000*g* for 10 min at 4 °C. The supernatant was collected, reserving 25 μl for protein analysis The remaining supernatant was subjected to Flag pulldown through incubation with 1:1 slurry M2-Flag magnetic beads (Sigma-Aldrich, M8823) for 2 h at 4°C. Beads were collected using a magnetic rack and two washes with modRIPA. Elution was performed by resuspending beads in 50 μl of 100 ng/μl Flag peptide (Sigma Aldrich, F4799) in 50 mM Tris-HCl and 150 mM NaCl. Tubes were rotated at 30 min at RT with the Flag peptide solution. Following elution and removal of beads samples were diluted with 1X Laemmli sample buffer, and resolved on a 4 to 20% polyacrylamide gel, followed by transfer to nitrocellulose membrane and western blotting.

### Nanoluciferase complementation assay

Nanoluciferase complementation was carried out with modifications as previously described (89). A total of 0.3 × 10^6^ HEK cells were seeded onto PDL-coated 6-well plates and incubated overnight at 37 °C with 5% CO_2_. The following day, cells were co-transfected with 600 ng Rap1GAP1a SmBiT with 200 ng HA-Gα_o_ LgBiT or Lyn LgBiT at a ratio of 1:3 and incubated for 24 h. After transfection, the media was aspirated, and cells were trypsinized and resuspended in 900 μl of Hank's balanced salt solution (HBSS) (Gibco, 14,175–095). The cell suspension was centrifuged at 250*g* for 5 min, and the supernatant was carefully removed. The intact pellet was resuspended in 2 ml HBSS, and cells from each condition were counted, re-pelleted, and resuspended in HBSS supplemented with 10 μM furimazine (Molnova, M23530) and 1% DMSO. A total of 50,000 cells were plated into individual wells of a 96-well plate (Greiner Bio-One, 655,083), with 4 technical replicates per condition. Following incubation with furimazine for 5 min at 37 °C, luminescence was measured in a Varioskan LUX plate reader at 37 °C for 5 min. Cells were subsequently collected from each well, pelleted by centrifugation at 600*g* for 3 min, and resuspended in 1X sample buffer for subsequent immunoblotting.

### Enhanced bystander BRET (ebBRET)/effector membrane translocation assay (EMTA)

ebBRET assays were carried out essentially as previously reported (45). 3.5 × 10^4^ cells were plated onto a PDL-coated 96-well plate. To measure G protein-dependent membrane recruitment, the luminescent donor Rap1GAP1a-RLucII, the fluorescent acceptors (rGFP-CaaX, rGFP-Giantin, rGFP-FYVE) and HA-tagged Gα_o_ (WT and the indicated mutants) were transfected at a ratio of 1:1:1. Reverse transfections were conducted with lipofectamine 2000. Immediately after seeding of the cells onto 96-well plates the DNA/Lipofectamine mixture was added. For receptor-dependent membrane recruitment, MOR-Flag and β_1_γ_2_ plasmids were introduced along with the G protein constructs and the aforementioned BRET pairs. Cells were maintained in 2% FBS media for 48 h following transfection.

Cells were rinsed with 1X PBS twice and incubated in 1X HBSS at room temperature (RT) prior to BRET measurements, 24 to 48 h after transfection, cells were rinsed with 1X PBS twice and incubated in 1X HBSS at room temperature (RT) prior to BRET measurements. For G protein-dependent membrane recruitment assays, cells were treated with 5 μM coelenterazine 400a (Goldbio, C-320–1) and luminescence was recorded for 10 min at RT using a BertholdTech TriStar2 multimode microplate reader with 400 nm (donor) and 515 nm (acceptor) filters. For receptor-based BRET experiments, cells were treated with 10 μM DAMGO (Tocris, 11,711) or morphine (Henry Schein, Melville, NY) and 5 μM coelenterazine 400a. Measurements were recorded for 20 min under the same conditions. Data from 4 technical replicates per condition were collected for the BRET measurements. BRET ratios were determined as the ratio of the light emitted by the GFP acceptor relative to the RLucII donor. Net BRET was calculated as BRET ratios from cells stimulated with DAMGO after subtraction of BRET ratios from cells treated with vehicle. Following the assay, the media was discarded, and cells were lysed with 1X sample buffer to verify protein expression *via* immunoblotting.

### RalGDS translocation assay

3.5 × 10^4^ HEK cells were plated into a PDL-coated 96-well plate. Each well was transfected with 35 ng of RalGDS-RlucII, 35 ng of rGFP-CaaX, 35 ng of HA-tagged Gα_o_ WT or mutant constructs, 35 ng of Flag-μ-opioid receptor, and 13 ng of Rap1GAP1a/Δ2-33Rap1GAP1a using Lipofectamine 2000 immediately after cell seeding. β1γ2 plasmids were co-transfected along with the G protein constructs. Following transfection, cells were maintained in 2% FBS media. The day after transfection, cells were serum-starved overnight or treated with 100 ng/ml pertussis toxin (PTX) (Sigma, P7208). On the day of the assay, cell media was carefully removed and incubated with 1x HBSS at RT. Following substrate addition in the presence and/or absence of agonist, BRET measurements were recorded. The BRET ratio was calculated as the light emitted by the GFP acceptor relative to the RlucII donor. Net BRET was derived from subtracting the basal BRET ratios from the BRET ratio of each well stimulated with DAMGO.

### Immunostaining

A293 (3 × 10^5^ cells) cells were plated into 6-well plates and transfected with plasmids encoding HA-Gα_o_. The day after transfection, cells were rinsed with 1X PBS, trypsinized, and pelleted by centrifugation at 1200 rpm for 2 min. After pelleting, cells were resuspended in DMEM and counted. A total of 3 × 10^4^ cells from each condition were plated on PDL-coated glass bottom dishes (D11030H, Matsunami). Approximately 16 to 18 h later, cells were rinsed with 1X PBS, fixed with 4% paraformaldehyde PFA (Electron microscopy sciences, 15,710) for 15 min at room temperature, and rinsed 3 times with 1x PBS. Blocking and permeabilization were performed using 1X PBS buffer supplemented with 0.2% Triton X-100 (Sigma, T8787) (also referred to as PBS-T) and 10% goat serum (Gibco, 16,210,064) for 1 h at RT. Following permeabilization, cells were incubated with rabbit monoclonal anti-HA and mouse monoclonal GM-130 primary antibodies at a ratio of 1:500 to 1000 overnight. The next day, cells were washed with PBS-T (5 min each wash) on a shaker, then probed with secondary antibodies diluted 10,000-fold in 2% goat serum -PBS-T. Following incubation with primary and secondary antibodies, nuclear staining was achieved with 4′,6′-diamidino-2phenylindole (DAPI). For imaging, cells were mounted on a 40 x Zeiss confocal lens or Leica DMi8 confocal microscope. DAPI fluorescence was detected using a 405 nm excitation filter, anti-mouse Alexa Fluor 488-conjugated secondary antibodies were visualized using a 488 nm excitation filter, and anti-rabbit Alexa Fluor 568-conjugated secondary antibodies were visualized using a 568 nm excitation filter.

### Western blotting

Samples prepared in 1X Laemmli sample buffer were boiled at 95 °C for 10 min. Boiled samples were resolved on a 4 to 20% Mini-protean TGXTM Gel (Bio-Rad, 4,561,094) and transferred onto a nitrocellulose membrane (GVS North America, 1,215,458). After successful transfer, the membrane blocked with 3% bovine serum albumin (BSA) (Fisher Scientific, BP1600) in TBS buffer supplemented with 0.1% Tween 20 (TBS-T) (0.1% Tween-20 in 20 mM Tris pH 7.5 + 150 mM NaCl) for 1 h on a shaker. Following blocking, membrane was incubated with primary antibodies diluted 1:500 to 1000 in TBS-T with 3% BSA and 0.1% sodium azide (NaN_3_) at RT for 2 h or 4 °C overnight. This was subsequently followed by four washes with TBS-T and probing with secondary antibodies diluted 10,000-fold in TBS-T-3%BSA at RT for 1 h with constant shaking. Antibody specificity was confirmed by comparing cells transfected with the indicated protein with untransfected cells. Finally, the membrane was washed, and protein bands were visualized using Li-Cor Odyssey CLX and analyzed using StudioLite software.

### Statistical analysis

All experiments were performed at least 3 times. Analysis was conducted with GraphPad Prism 7.0 (GraphPad). All data are represented as means ± SEM. Analysis was carried out with *t*-tests, one-way ANOVA, and two-way ANOVA with Tukey's multiple and Dunnett's comparisons test (∗*p* < 0.05 ∗∗*p* < 0.01, ∗∗∗ < 0.001, ∗∗∗∗<0.0001) as indicated in the figure legends.

## Data availability

All data are contained within the manuscript and supporting information. Mass Spectrometry Data will be deposited in the PRIDE proteomics database. The structures used in this study were accessed from the Protein Data Bank (PDB). Any plasmids and recombinant proteins described in this study are available from the corresponding author upon reasonable request subject to a completed Materials Transfer Agreement.

## Supporting information

This article contains supporting information.

## Conflict of interest

The authors declare that they have no conflicts of interest with the contents of this article.

## Supporting information

G protein alpha o TurboID screen
