# Supplementary material for: GoLoco/GPR motif-dependent regulation of Rap1GAP1 by Gαo is disrupted by Gαo encephalopathy variants
Source: J Biol Chem. 2025 Jul 2;301(8):110446. doi: 10.1016/j.jbc.2025.110446 (PMC12329598; doi:10.1016/j.jbc.2025.110446)

**Supporting Information**

**GoLoco/GPR motif-dependent regulation of Rap1GAP1 by Gα_o_ is disrupted by Gα_o_ encephalopathy variants**

Nathalie L. Momplaisir^1^, Naincy R. Chandan^1^, Beiyun Wang^1^, Elaine Qu^1^, and Alan V. Smrcka^1#^

^1^Department of Pharmacology, University of Michigan Medical School, Ann Arbor, MI

Supplemental Figures 1-6

**
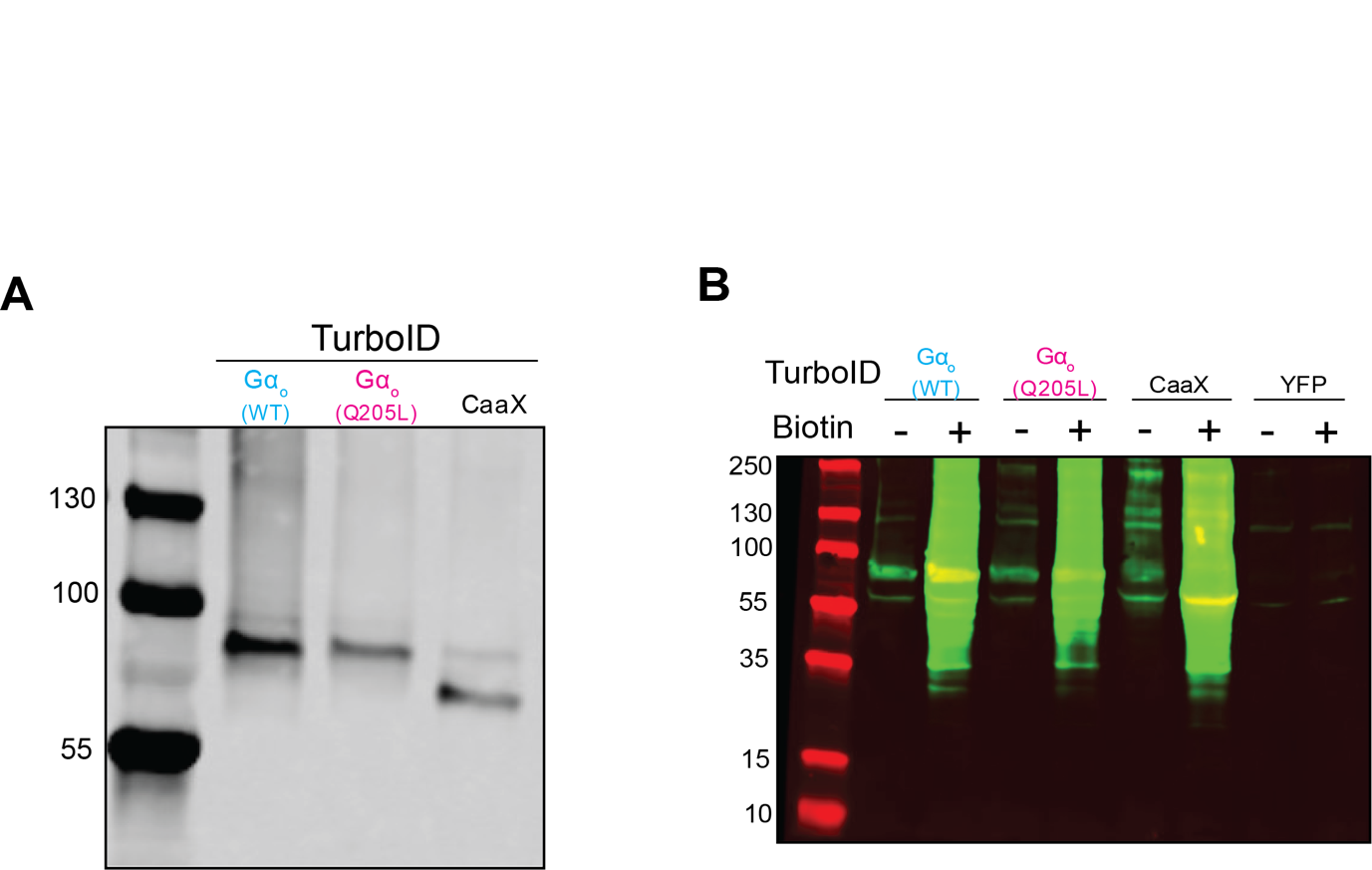
**

**Figure S1 Validation of proximity-based biotinylation by TurboID-fused Gα_o_ and CaaX**

**(A)** Differentiated PC12 cells were transduced with TurboID-Gα_o_^(WT)^, TurboID-Gα_o_^(Q205L)^, and TurboID-CaaX. Cell lysates were immunoblotted with a V5 antibody to confirm the expression of TurboID-Gα_o_ and TurboID-CaaX. **(B)** After biotinylation, cell lysates were probed with streptavidin antibody to assess biotinylation across the transfection conditions by TurboID-Gα_o_ and TurboID-CaaX. N=2

**Figure S2 Gene ontology annotation of TurboID-Gα_o_^(WT)^ enriched candidates**

**(A)** Filtering strategy for proteins enriched by TurboID-Gα_o_^(WT)^. **(B)** Gene ontology analysis of the prominent cellular localization of TurboID-Gα_o_^(WT)^ enriched candidates. **(C)** Representative biological processes from GO analysis of TurboID-Gα_o_^(WT)^ enriched proteins.


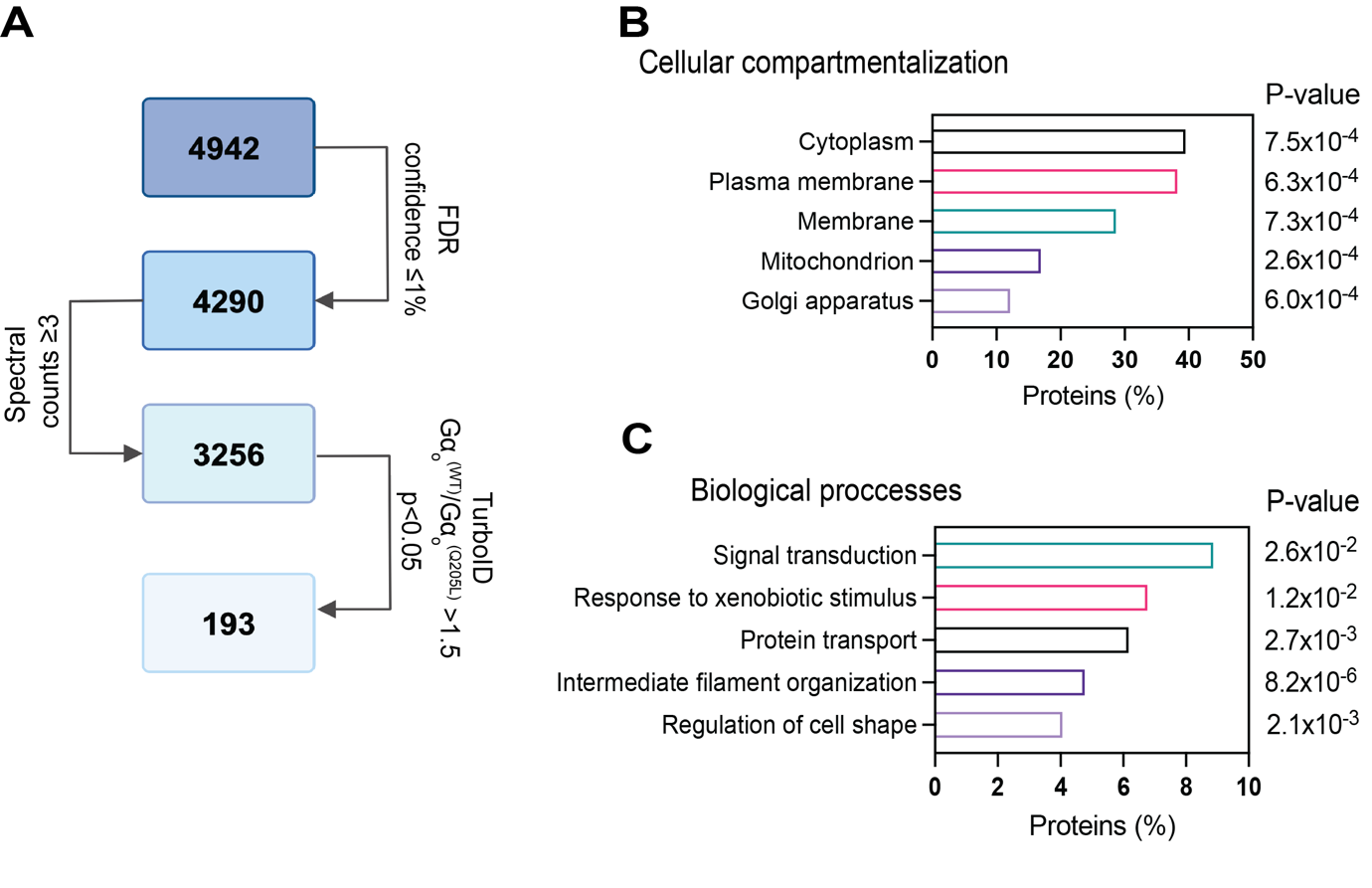


**Figure S3 Sequence alignment of protein coding Rap1GAP1a variants**

Alignment of human Rap1GAP1 variants. Stars (*) represent identical amino acids across all variants. Identifiers for each variant were obtained from Uniprot and are shown to the left.


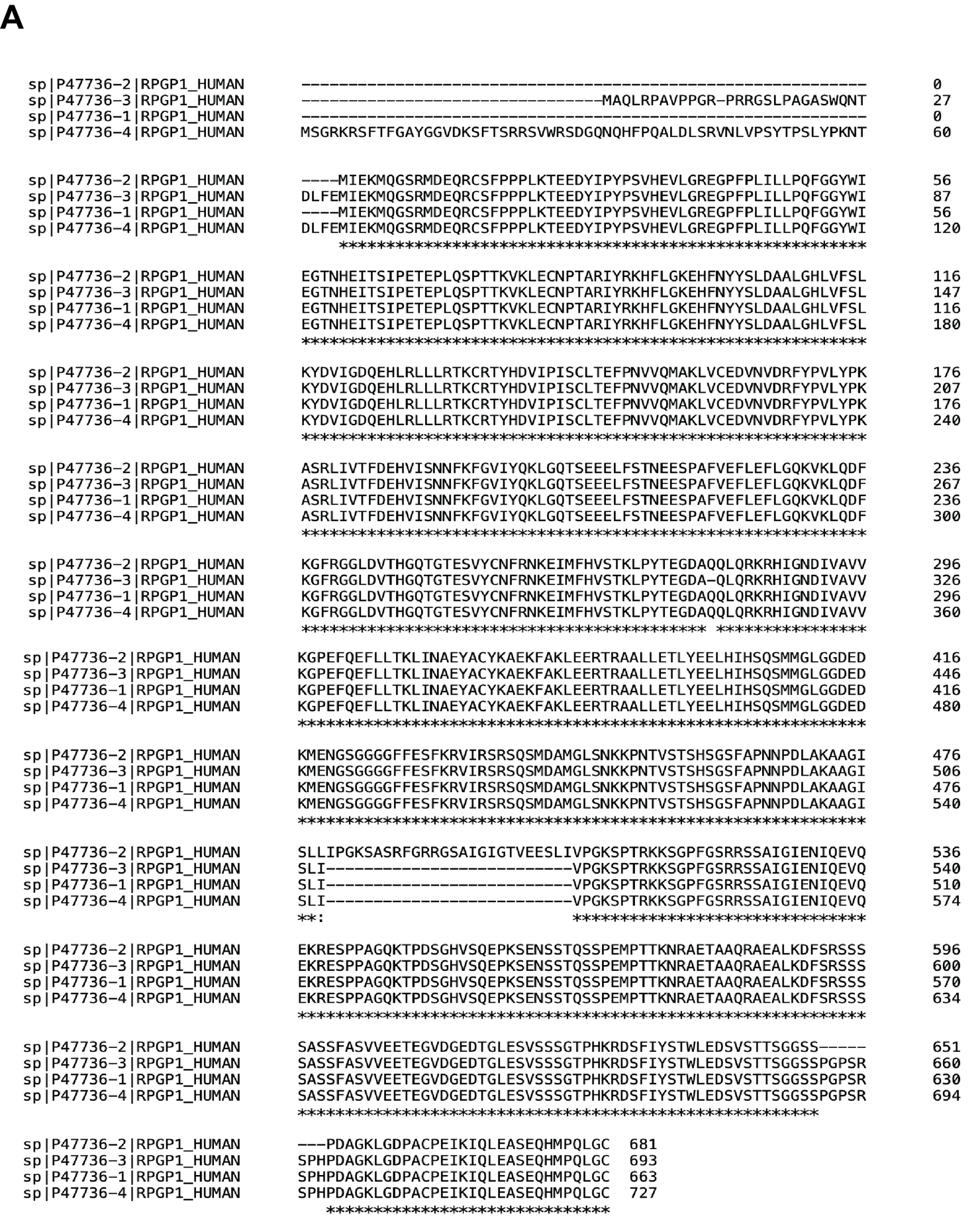

**Figure S4 PTX sensitivity of Rap1GAP1a translocation to the PM and dual localization of Gα_o_ to PM and Golgi regardless of activation state**

**(A)** A293 cells were transfected with μ-opioid receptor, Gα_o_, Rap1GAP1a-RlucII and rGFP-CaaX. 24 hours after transfection, cells were starved with a subset of cells pre-treated with PTX overnight. The next day, BRET was measured after the addition of coelenterazine 400a. Data represent means ± SEM of three independent experiments (**p<0.01, ***p<0.001, calculated using two-way ANOVA with Tukey’s multiple comparisons test). **(B)** Confocal images of A293 cells co-transfected with HA- Gα_o_ (red) and the cis Golgi marker (GM130) (green). Scale bar = 10 μM.

**Figure S5** **Expression levels of Rap1GAP1a and Rap1GAP1b in the presence of Gα_o_**

**(A)** Representative western blots of indicated transfected constructs following EMTA in Fig. 4B and cell lysis. **(B)**. The indicated Rap1GAP1b constructs were co-transfected with Gα_o_. Following EMTA, cells were lysed and run on an SDS-PAGE. Western blots are representative of BRET assays from Fig. 4D performed three times.


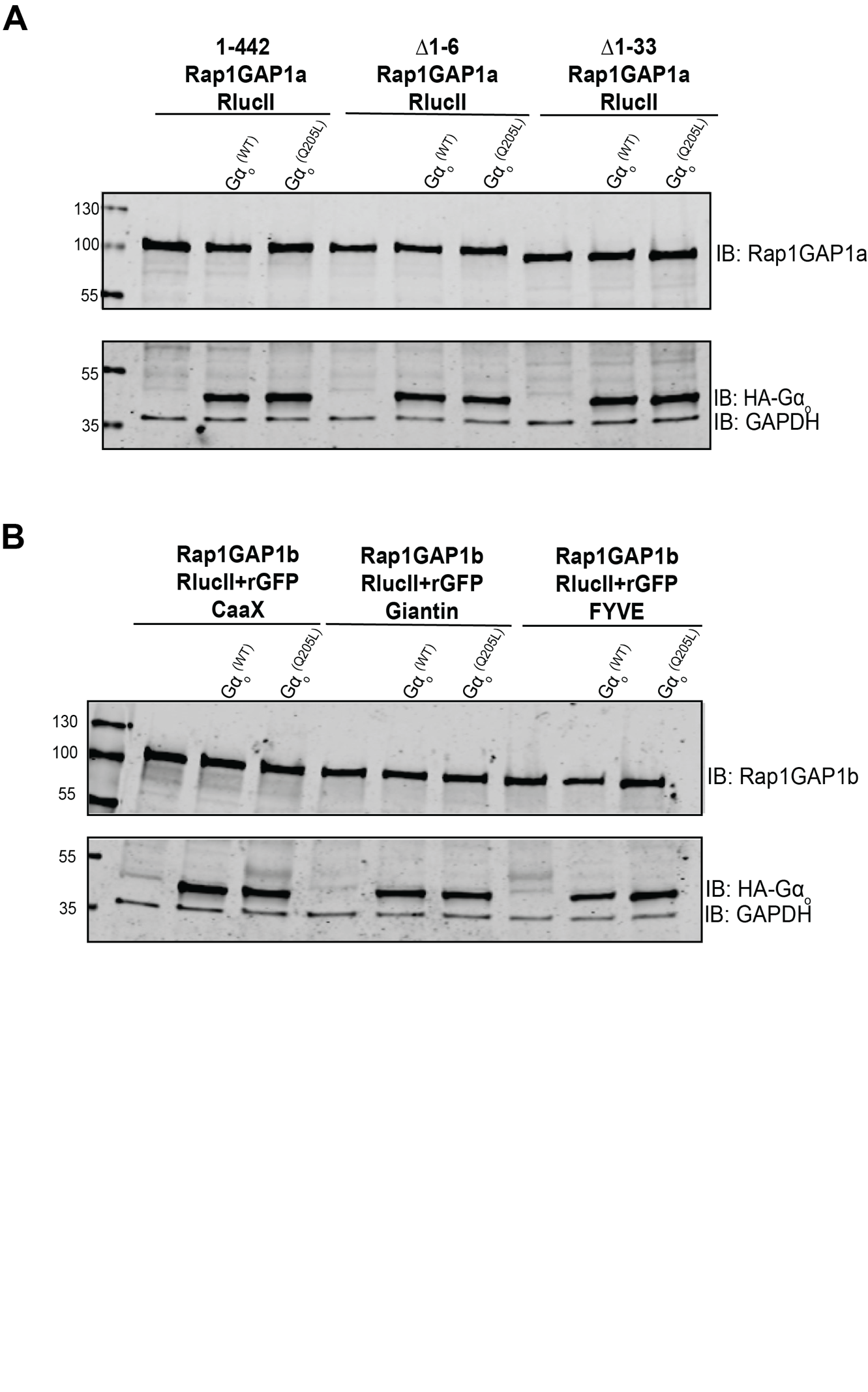


**Figure S6 Characterization of RalGDS biosensor**

**(A)** Time-course for EGF-dependent activation in HEK cells transfected with RalGDS-RlucII and rGFP-CaaX. After EGF stimulation, RalGDS recruitment to the plasma membrane was monitored over 20 mins. Representative BRET data, performed in 4 technical replicates, are represented as BRET ratio and means ± SEM. **(B)** HEK cells transfected with RalGDS-RLucII and rGFP-CaaX, with or without Rap1GAP1a were treated with EGF for 10 mins. BRET ratios are normalized to vehicle and are shown as means ± SEM of 4 independent experiments, each performed in 4 technical replicates (*p<0.05, **p<0.01, calculated using two-way ANOVA with Tukey’s multiple comparisons test).


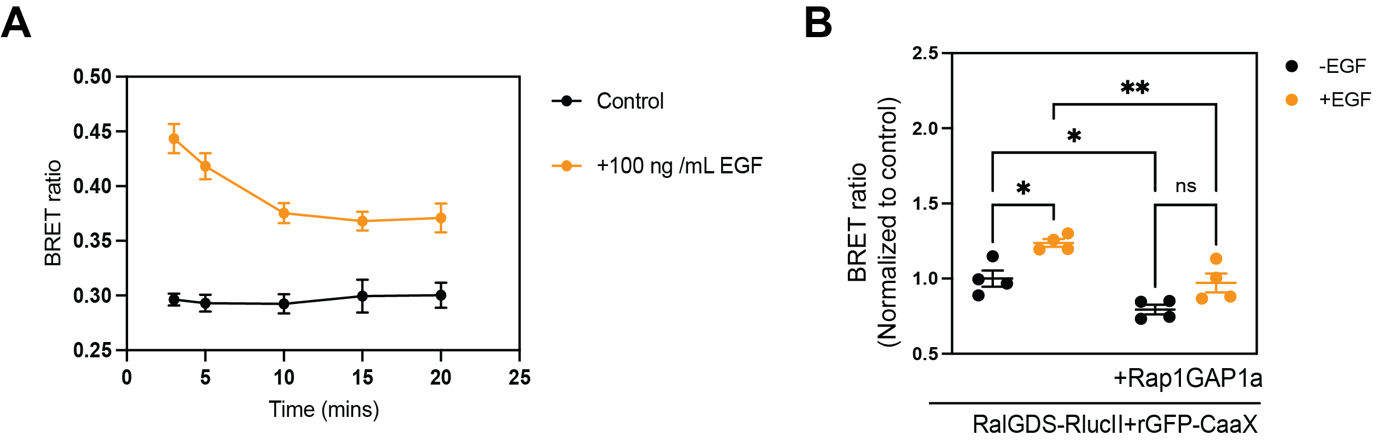

Supplement: Supplemental Information [file mmc2.docx]
